# Recent Progress on NIR-II Photothermal Therapy

**DOI:** 10.3389/fchem.2021.728066

**Published:** 2021-07-29

**Authors:** Yunguang Zhang, Siyu Zhang, Zihan Zhang, Lingling Ji, Jiamei Zhang, Qihao Wang, Tian Guo, Simin Ni, Ru Cai, Xiaoyu Mu, Wei Long, Hao Wang

**Affiliations:** ^1^ School of Science, Xi’an University of Posts and Telecommunications, Xi’an, China; ^2^ Tianjin Key Laboratory of Brain Science and Neuroengineering, Academy of Medical Engineering and Translational Medicine, Tianjin University, Tianjin, China; ^3^ Tianjin Key Laboratory of Radiation Medicine and Molecular Nuclear Medicine, Institute of Radiation Medicine, Chinese Academy of Medical Sciences and Peking Union Medical College, Tianjin, China

**Keywords:** photothermal therapy, cancer therapy, photothermal conversion, nanomaterials, NIR-II

## Abstract

Photothermal therapy is a very promising treatment method in the field of cancer therapy. The photothermal nanomaterials in near-infrared region (NIR-I, 750-900 nm) attracts extensive attention in recent years because of the good biological penetration of NIR light. However, the penetration depth is still not enough for solid tumors due to high tissue scattering. The light in the second near-infrared region (NIR-II, 1000-1700 nm) allows deeper tissue penetration, higher upper limit of radiation and greater tissue tolerance than that in the NIR-I, and it shows greater application potential in photothermal conversion. This review summarizes the photothermal properties of Au nanomaterials, two-dimensional materials, metal oxide sulfides and polymers in the NIR-II and their application prospects in photothermal therapy. It will arouse the interest of scientists in the field of cancer treatment as well as nanomedicine.

## Introduction

Cancer is also called malignant tumors, a remarkable feature of which is the rapid formation of abnormal cells and metastasis. Researches show that cancer is a leading cause of death (Li et al., 2017b), around one in six deaths worldwide are caused by cancer. Researchers are committed to exploring effective ways to treat cancer., Among various therapeutic methods, photothermal treatment (PTT) has attracted wide attention (Sun et al., 2019a; Zhu et al., 2019; Wen et al., 2020a; Zhen et al., 2021). PTT is a method of killing cancer cells using materials with high photothermal conversion efficiency and converting light energy into heat energy under the irradiation of an external light source (Hu et al., 2020). Recent studies have shown that near-infrared light has a better biological tissues penetration, thus near-infrared absorption materials for PTT show great development potential for clinical diagnosis and disease treatment (Tang et al., 2019a; Li et al., 2020b). PTT treatment can relieve the pain of patients, shorten the treatment time, improve treatment effect and reduce the toxicity. However, the applications of this technique is further limited due to its relatively shallow tissue penetration depth in the traditional NIR-I region (Lyu et al., 2019). Currently, the biologically-related applications of NIR-II has aroused great interest, which can be used for NIR-II PTT and NIR-II fluorescence imaging (Liu et al., 2019; Suo et al., 2019; Chen et al., 2021). It has the advantages of low light scattering, high spatial resolution and deep tissue penetration (Yang et al., 2017a).

In particular, with the help of therapeutic materials, the PTT of NIR-II can be combined with a variety of treatment methods to improve the therapeutic effect (Lyu et al., 2019). Briefly, cell death occurs when the local tissue temperature is 42–46°C for 10 min. When treated with PTT, the cells will die rapidly when the temperature exceeds 60 °C (Li et al., 2020b). The key to the applications of the NIR-II absorption materials in PTT is that the materials should have good photothermal conversion efficiency, low biological toxicity and easy functionalization. Studies show that Au nanomaterials (Deng et al., 2019; Song et al., 2019; Wang et al., 2020c; Jia et al., 2020), metal sulfur oxides (Lin et al., 2020a; Sun et al., 2021) such as Ag_2_S (Yang et al., 2017b) and MoO_2_ (Guo et al., 2020), two-dimensional nanomaterials such as MoS_2_ (Shi et al., 2020), niobium carbide (MXene) (Lin et al., 2017b) and SnTe (Zhang et al., 2019a), and polymers (Sun et al., 2019a) such as semiconductor polymer nanoparticles SPN_1_ (Jiang et al., 2018b), SPN_2_ (Yin et al., 2020), SPN_3_ (Cao et al., 2018b) can be applied for NIR-II PTT. By modifying the photothermal materials, researchers can improve their photostability, chemical stability, biocompatibility, and endow them with some new functions, such as targeting and drug delivery.

**FIGURE 1:**
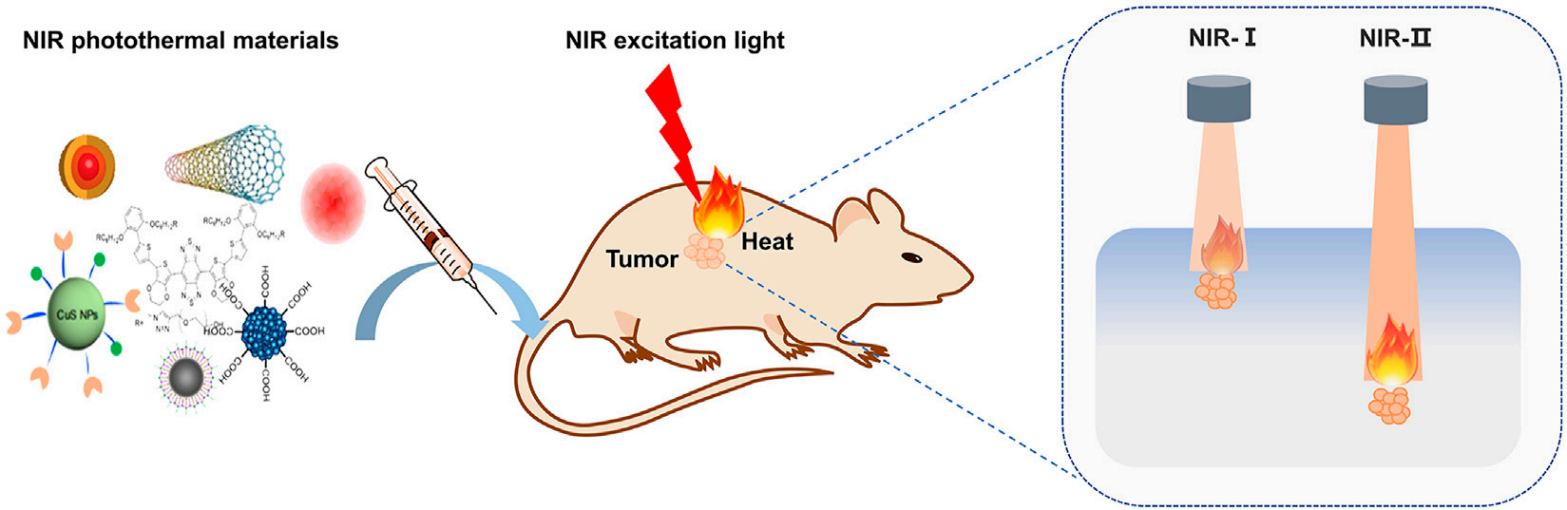
Schematic of NIR photothermal therapy. The photothermal materials were administrated and enter the tumor cells, then excited by NIR light and transform the light energy into heat to ablate tumor cells. The lights in NIR-I and NIR-II regions have different penetration depths through tissues, and NIR-II excitation is superior to its counterpart and promised better prospect for the treatment of deep tumors.

Gold nanomaterials, two-dimensional materials, metal sulfide oxide materials, polymers, carbon nanomaterials and NIR-II dyes have been being investigated as PTT agents both in NIR-I and NIR-II regions. The NIR-II PTT materials showed a better prospect for the treatment of deep tumors, which is favored by the deep tissue penetration of NIR-II excitation light. In the present work, we summarized the NIR-II optical absorption materials, analyzed the photothermal conversion efficiency of different materials and concluded their biological applications such as cancer treatment. Some prototypical materials for use in NIR-II PTT, the process of PTT, and the difference between NIR-I and NIR-II are shown in Figure 1.

## NIR-II Absorbing Materials

### Gold Nanomaterials

**FIGURE 2:**
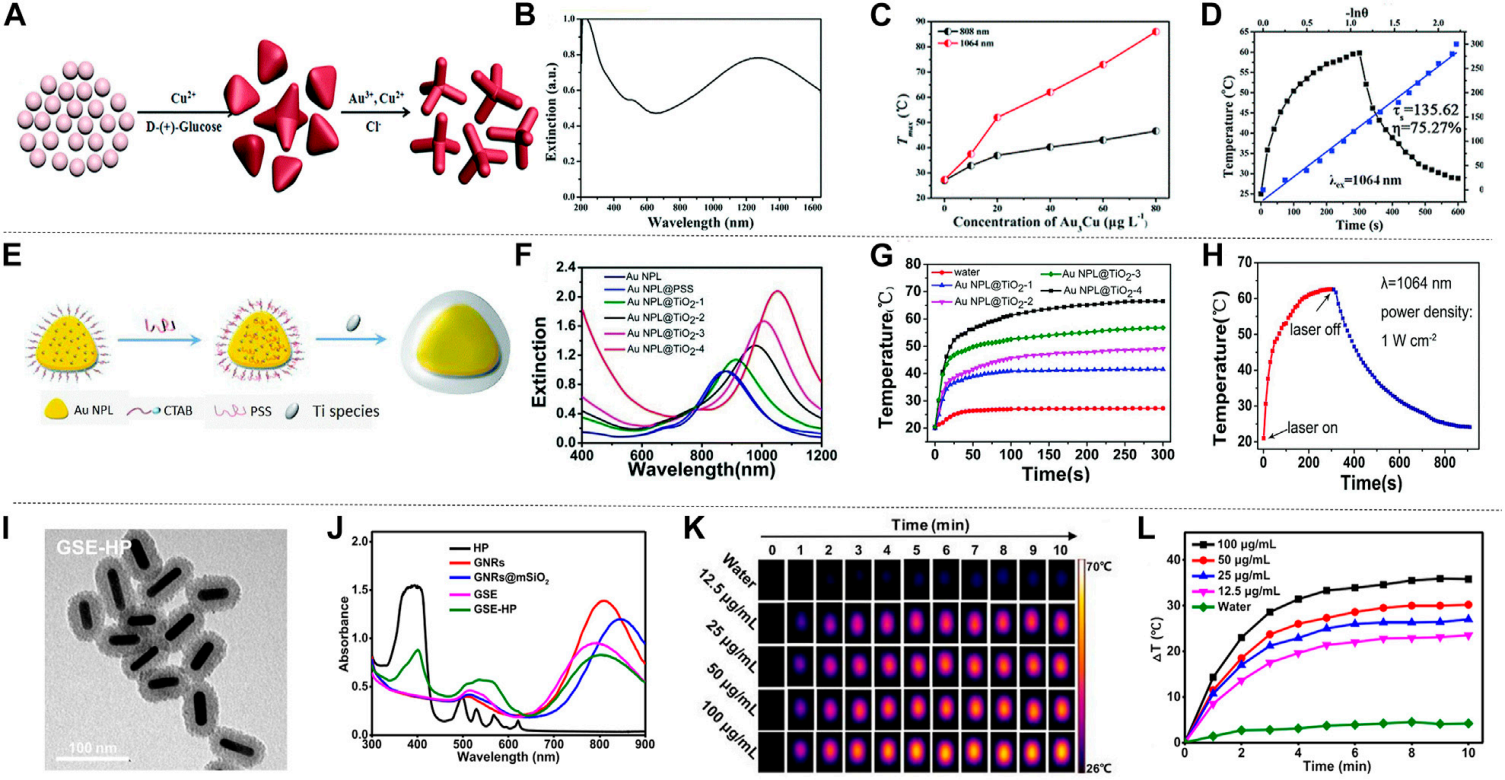
The photothermal properties of Au nanomaterials. **(A)** Numerical control growth of tetrapods. **(B)** Ultraviolet-visible-NIR (UV-vis-NIR) extinction spectrum of TPNCs. **(C)** The relationship between the concentration of TPNCs after irradiating Au_3_Cu@PEG TPNC with 808 and 1,064 nm laser for 5 min. **(D)** The photothermal conversion efficiency of Au_3_Cu@PEG TPNCs under 1,064 nm laser irradiation, the blue line is the heat transfer time constant of the system determined by linear time data during cooling (reproduced from (Wang, at al., 2018) with permission from Royal Society of Chemistry). **(E)** Schematic diagram of the synthesis of AuNPL@TiO_2_. **(F)** Extinction spectra of conventional gold nanoplate samples and gold nanoplate samples coated with different thickness shells. **(G)** The temperature changes of AuNPL@TiO_2_ with different thicknesses of shell layer within 5 min of irradiation with a laser with an intensity of 1,064 nm (1 W cm^−2^), in which water is the blank control group. **(H)** Schematic diagram of the photothermal effect of AuNPL@TiO_2_-4 with a shell thickness of 68 nm irradiated with a 1,064 nm laser (reproduced from (Gao et al., 2019) with permission from Royal Society of Chemistry). **(I)** Transmission electron microscope image of GSE-HP. **(J)** Absorption spectra of GSE-HP and contrast materials. **(K)** Thermal image of GSE-HP. **(L)** Use 808 nm laser with a power density of 0.8 W cm^−2^ to irradiate GSE-HP with different concentrations, and the corresponding heating curve (reproduced from (Luo et al., 2020) with permission from American Chemical Society).

In recent years, gold-based nanomaterials have been widely utilized in various fields. Gold nanoparticles have the characteristics of high electron density and can change the scattering angle of certain lasers, making them available for the application of nano-gold labeling and fluorescence labeling. The above-mentioned applications are inseparable from the excellent compatibility of gold nanomaterials with biological tissues. Further, multiple reports proposed the application of gold nanomaterials in PTT. Near-infrared localized surface plasmon resonance (LSPR) peak tunability (Li et al., 2016), excellent photothermal conversion, good stability and easy surface functionalization are the most important characteristics of gold-based nanomaterials in photothermal treatment. They are the most representative nanostructures and are recognized as an idealized light-to-heat converter. Based on these features, the use of gold nanomaterials as raw materials provides an additional solution for the design of future photothermal agents. The currently reported methods for preparing gold nanomaterials are mainly seed-mediated growth methods (Plan Sangnier et al., 2019). Wang et al. (2018c) prepared a kind of Au_3_Cu tetrapod nanocrystals (TPNCs) by this method. In the process of preparing TPNCs, 3 nm Au seeds were added to the solution containing HAuCl_4_•3H_2_O, CuCl_2_•2H_2_O, D-(+)-glucose, 1-hexadecylamine and NH_4_Cl, and then heated to 100°C, and maintained in a nitrogen-filled environment for 40 min. Figure 2A shows the NC growth process of TPNCs at different stages. The average arm length of the produced TPNCs (the arm length of the tetrapod) is about 22.8 ± 4.6 nm. The extinction spectrum of TPNCs was measured with an ultraviolet-visible spectrophotometer. TPNCs show strong broad peaks in the 700–1,400 nm spectral region, and completely contain the NIR-I and NIR-II, as shown in Figure 2B. Generally, thiol polyethylene glycol (PEG) amine (HS-PEG-NH_2_), the water-soluble fluorescent dye Cy5 labeled polyethylene glycol derivatization reagent (HE-PEGNH-Cy5) and derivatization reagent of mercaptopolyethylene glycol folic acid (HS-PEGNH-FA) can improve the water solubility and biocompatibility of nanomaterials. Therefore, to improve the physiological stability and specific targeting ability for cancer treatment, after TPNCs were obtained, the surfaces of HS-PEG-NH_2_, HE-PEGNH-Cy5, HS-PEGNH-FA and TPNCs were combined to obtain Au_3_Cu@PEG TPNCs.

Generally, there are two main factors that determine the photothermal performance of nanomaterials: photothermal conversion efficiency and extinction coefficient. The theoretical calculation showed the mass extinction coefficient of Au_3_Cu@PEG TPNCs is 53.0 L g^−1^ cm^−1^ at 1,064 nm. Compared with the previously studied inorganic photothermal agents, Au_3_Cu@PEG TPNC has embodied its superiority. The Au_3_Cu@PEG TPNC was treated with 808 and 1,064 nm laser radiation respectively. Obviously, the temperature increment and the therapeutic effect of radiation treatment at 1,064 nm was much higher than that of 808 nm, indicating that Au_3_Cu@PEG TPNC has higher absorption intensity under 1,064 nm laser radiation (Figure 2C). Moreover, to evaluate the photothermal characteristics of Au_3_Cu@PEG TPNC comprehensively, the photothermal conversion efficiency was also measured. As shown in Figure 2D, the photothermal conversion efficiency of Au_3_Cu@PEG TPNC is 75.27% when irradiated with a 1,064 nm laser, which is much higher than the previous report. Song et al. (2019) also used a seed-mediated growth method, using gold chloride, ascorbic acid, and Cetyl trimethyl ammonium Chloride (CTAC) as a growth solution, synthesized a kind of gold nanostars with good near-infrared SERS activity and photothermal effect. The gold nanostar has been further designed as a multifunctional nano-agent. Gao et al. also reported the heterostructure of titanium dioxide-coated gold nanosheets (AuNPL@TiO_2_) synthesized using a seed-mediated growth method (Figure 2E; Gao et al., 2019). This structure has a controllable shell thickness, which can be adjusted for different clinical applications. Under scanning electron microscopy and transmission electron microscopy, AuNPLs showed good dispersion in water and uniform shape, with a particle size of about 118 nm. As the thickness of the shell increased, the nanostructures were divided into gold nanosheet 1 (AuNPL@TiO_2_-1), gold nanosheet 2 (AuNPL@TiO_2_-2), gold nanosheet 3 (AuNPL@TiO_2_-3) and gold nanosheet 4 (AuNPL@TiO2-4), the thickness of the titanium dioxide shell can be changed by adjusting the concentration of sodium bicarbonate. From the extinction spectra of AuNPL@TiO_2_ with different shell thicknesses, it can be seen that as the wavelength increases, the thicker the shell of the material, the higher the extinction index, and the more effective it is to reduce the heat loss (Figure 2F). To reveal the photothermal characteristics of the AuNPL@TiO_2_ structure at the NIR-II wavelength, AuNPL@TiO_2_ with different shell thicknesses was irradiated under a 1,064 nm laser for 5 min (Figure 2G). In addition, the Roper’s method was used to calculate the AuNPL@TiO_2_-4 photothermal conversion efficiency. When continuously irradiated by a 1,064 nm laser for 5 min and then the laser is turned off, the temperature-time function recorded is shown in Figure 2H. AuNPL@TiO_2_-4 showed an exceptionally excellent light-to-heat conversion efficiency of 42.05%. This is attributed to the closer match between the strong plasma absorption peak of the AuNPL@TiO_2_-4 structure and the wavelength of the 1,064 nm laser. Besides, the red shift effect caused by the titanium dioxide shell around the gold nanoparticles may also play an important role in the photothermal conversion of the near-infrared region. Under the same conditions, the gold nanosheet four irradiated with 1,064 nm laser showed a stronger photothermal effect than that irradiated with 808 nm laser. Similarly, Yang et al. (2020) also prepared a gold nanorod with a similar aspect ratio and LSPR peak with adjustable width and length, which has a strong photothermal conversion efficiency under 980 nm laser irradiation. New nanomaterials with large mesopores have attracted much attention because they can achieve the adsorption and separation effects that are difficult to complete with traditional macromolecular materials. Luo et al. (2020) discovered a gold nanorods (GNR) nano sensitizer coated with large mesoporous silica, and combined with hematoporphyrin (HP) to generate a new type of nano sensitizer (GNRs@mSiO_2_−EuBA/HP (GSE-HP). The transmission electron microscope image of GSE-HP is shown in Figure 2I. This new type of nano-sensitizer (GSE-HP) was also synthesized using a seed-mediated growth method, and its length and width are 57.1 ± 2.9 and 15.7 ± 1.4 nm, respectively. GSE-HP contains a layer of silica coating to increase light transmittance, causing a redshift of absorbance from 810 to 846 nm, as shown in Figure 2J. GSE-HP has very strong light absorption at 806 nm, and exhibits obvious temperature changes under laser irradiation. The temperature of the GSE-HP solution rapidly increased by 30 °C within 3 minutes after being irradiated, as shown in Figures 2K,L. The light-to-heat conversion efficiency (η value) of GSE-HP is 38.4%. The photothermal conversion ability of GSE-HP hardly decreased, which proved its strong photothermal stability. Wang et al. (2020c) explored a special liposome template-oriented route to synthesize a new type of gold nanoframe with large mesopores (40 nm). The average hydrodynamic diameter of this gold nanoframe is 140.2 ± 3.2 nm. Under the irradiation of 1,064 nm laser, the photothermal conversion efficiency of the gold nanoframe is 23.9%. Liu et al. (2011) used a thin gold nanoshell and a monodisperse mesoporous silica nanocore to synthesize a multifunctional gold nanoshell with a unique structure of movable core and mesoporous shell. This shell layer has excellent thermal stability, chemical stability and mechanical stability. All the above-mentioned gold nanomaterials show different degrees of advantages as photothermal agents. In the future, it is vital to seek simple, efficient, low-cost and green ways to prepare the above-mentioned gold nanomaterials.

### Two-Dimensional Materials

**FIGURE 3:**
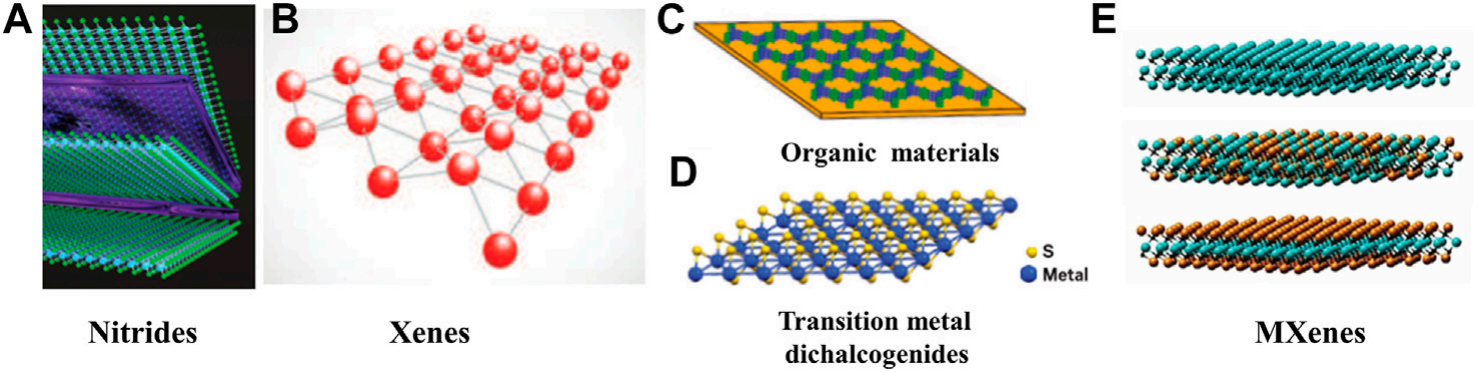
Classification of two-dimensional materials. **(A)** Nitrides. **(B)** Xenes. **(C)** Orangic materials. **(D)** Transition metal dichalcogenides (reproduced from (Jacoby, 2017) with permission from Royal Society of Chemistry). **(E)** MXenes (reproduced from (Huang et al., 2018) with permission from Royal Society of Chemistry).

Two-dimensional materials (2D materials) are favored by researchers because of their unique properties such as ultra-thin, huge surface area, and photothermal conversion performance under NIR irradiation (Xie et al., 2016; Xing et al., 2017). 2D materials can be divided into five categories, namely nitrides, Xenes, organic materials, transition metal dichalcogenides (TMDs) (He et al., 2019), and MXenes (Figures 3A–E) (Jacoby, 2017; Huang et al., 2018).

**FIGURE 4:**
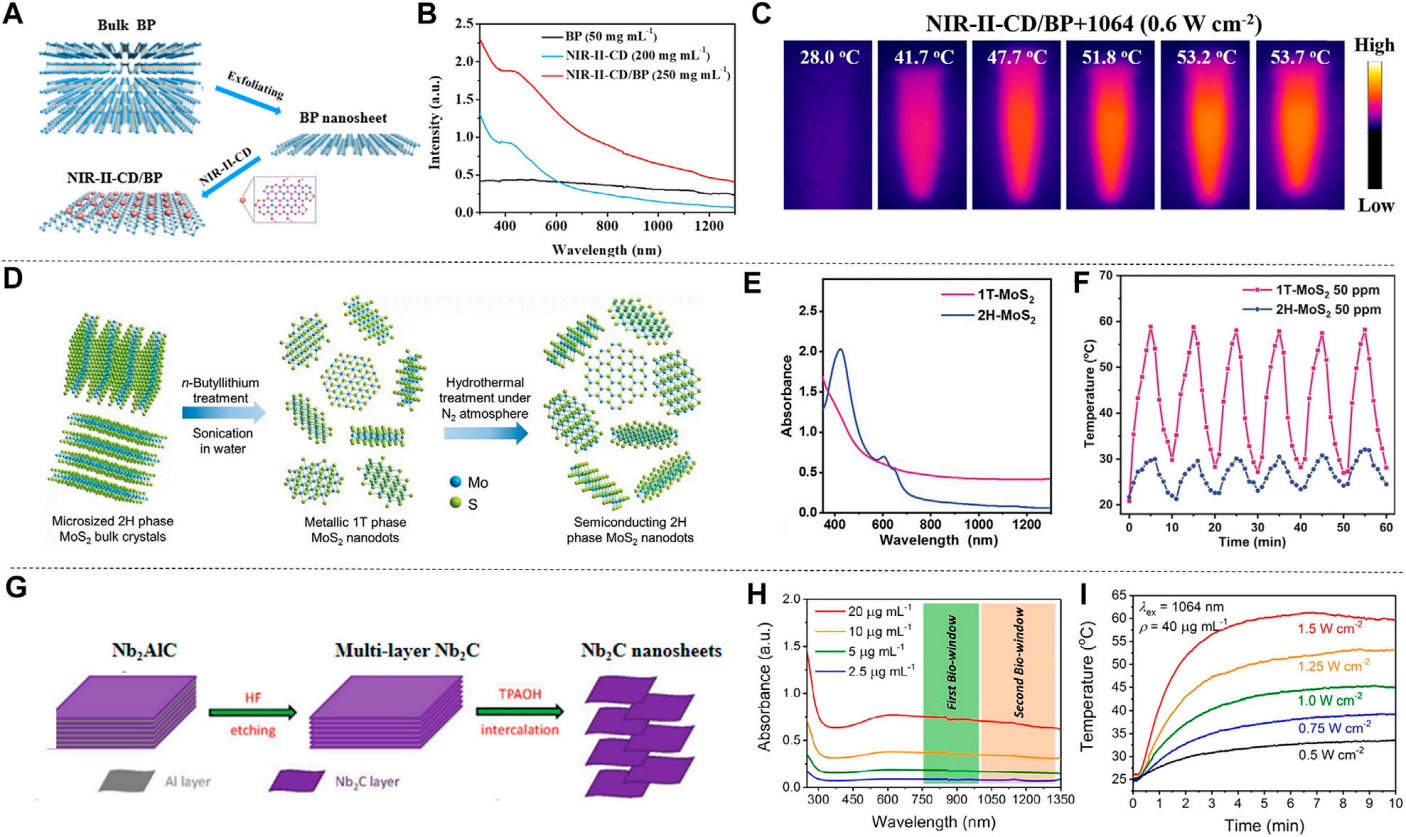
Examples of 2D material synthesis and optical properties. **(A)** Synthesis process of NIR-Ⅱ-CD/BP. **(B)** Absorption spectra of BP NSs, NIR-Ⅱ-CD and NIR-Ⅱ-CD/BP from UV to NIR-Ⅱ regions. **(C)** Intuitive temperature image of NIR-Ⅱ-CD/BP solution under 1,064 nm (0.6 W cm^−2^) irradiation (reproduced from (Geng et al., 2019) with permission from American Chemical Society). **(D)** Synthesis process of ultra-thin single layer 1T-MoS_2_ and 2H-MoS_2_ nanodots. **(E)** UV-vis-NIR spectra of 1T-MoS_2_ and 2H-MoS_2_ nanodots. **(F)** The temperature change of 1T-MoS_2_ and 2H-MoS_2_ nanodots solution after six times of laser on/off (heating/cooling) (reproduced from (Zhou et al., 2020) with permission from Wiley-VCH Verlag). **(G)** Preparation process of Nb_2_C NSs. **(H)** UV-vis-NIR absorption spectra of Nb_2_C NSs with different concentrations. **(I)** Temperature change of Nb_2_C NSs irradiated with different power density 1,064 nm laser (reproduced from (Lin et al., 2017) with permission from American Chemical Society).

Xenes atoms represented by black phosphorus, silicene, and germanene are arranged in a honeycomb shape. In virtue of the simple chemical composition, they are easier to synthesize than other multi-element 2D materials, and have good physical, chemical and optical properties. Geng et al. (2019) loaded black phosphorus nanosheets (BP NSs) with NIR-II-responsive carbon dots (NIR-II-CDs) to form NIR-II-CD-passivated BP (NIR-II-CD/BP), BP NSs and NIR-II-CDs were prepared by liquid exfoliation method (Wang et al., 2018b) and microwave reaction, respectively (Figure 4A). Due to the accumulation of free carriers on the BP NSs in the NIR-II-CD/BP hybrid, they may induce the LSPR effect to enhance the near-infrared absorption (Ding et al., 2014; Cao et al., 2018a). The absorption spectra of BP NSs, NIR-II-CD and NIR-II-CD/BP solutions are shown in Figure 4B. It is observed that the absorbance peak of NIR-II-CD/BP appears at 1,064 nm, reaching 0.590, while the sum of the absorbance of the original BP NSs and NIR-II-CD is only 0.425, indicating that the absorbance of NIR-II-CD/BP has been improved, rather than a simple physical mixture of NIR-II-CD and BP NSs. After 1,064 nm laser irradiation, the temperature of NIR-II-CD/BP increased by 25.7°C, and finally reached 53.7°C (Figure 4C), showing excellent photothermal conversion ability; while the temperature of NIR-II-CD and BP NSs only increased to 48.2°C and 44.3°C under the same conditions. In addition, NIR-II-CD/BP and BP NSs were irradiated with 1,064 nm and 808 nm lasers several times, and they were turned off after a certain period of time. The temperature of NIR-II-CD/BP showed no significant change after each photothermal conversion, and the photothermal stability was confirmed. According to calculation results, the photothermal conversion efficiency of NIR-II-CD/BP has been improved to 61.4% comparing with the original BP NSs which is only 28.4%. Wang et al. (2020a) prepared 2D silicene NSs through a topological chemical synthesis method of gentle oxidation and exfoliation, and studied the photonic drug-delivery nanoplatform based on 2D silicene nanosheets (DOX@silicene-BSA NSs), which show strong light absorption and conversion capabilities in both NIR-I and NIR-II biowindows. Duan et al. (2020) designed MnO_x_@silicene-BSA (MS-BSA) based on 2D silicene semiconductor nanosheets. 2D silicon exhibits unique physical and chemical advantages due to its rare low-buckled topography, and BSA was used for surface functionalization to further realize colloidal stability, improving photothermal conversion ability and photothermal stability. TMDs represented by MoS_2_ and WS_2_, have a stacked layered structure, which can crystallize into different crystal phases, and more particularly, different phase states of transcranial magnetic stimulation can show completely different characteristics (Zhou et al., 2020b). Zhou et al. (2020b) prepared single-layer metal 1T-phase MoS_2_ (1T-MoS_2_) nanodots by chemical lithium intercalation method from micron-sized 2H-phase MoS_2_ (2H-MoS_2_) microcrystals (Figure 4D), and compared the absorption spectrum, photothermal characteristics and other properties of 1T-MoS_2_ and 2H-MoS_2_. The UV-vis-NIR spectra of 1T-MoS_2_ and 2H-MoS_2_ are shown in Figure 4E, 1T-MoS_2_ has no obvious absorption peak due to its metallic properties, and it exhibits optical absorption in the visible region to the infrared regions. After six times of 1,064 nm laser on/off (heating/cooling), the maximum temperature of the two photothermal conversions did not change significantly, and the temperature of 1T-MoS_2_ was significantly higher than that of 2H-MoS_2_, reflecting the excellent light response ability and photothermal stability of 1T-MoS_2_ (Figure 4F). In addition, irradiating 1T-MoS_2_ nanodots and 2H-MoS_2_ nanodots with laser for 8 min, the temperature of 1T-MoS_2_ nanodots solution increased by 43.0°C, while the temperature of 2H-MoS_2_ nanodots solution only increased by 12.4°C, indicating that 1T-MoS_2_ nanodots have the ability to quickly convert near-infrared light energy into heat energy. In addition, the temperature changes of 1T-MoS_2_ and 2H-MoS_2_ solutions with different concentrations under 1,064 nm laser irradiation were measured, and the increment of the temperature boosted with increasing concentration. It can be seen that 1T-MoS_2_ not only exhibits strong light absorption capacity in the NIR-II window, but also shows unique photothermal conversion ability which makes it an excellent candidate material for photothermal reagents. The 2D material MXenes contains transition metal carbides, nitrides and carbonitrides (Naguib et al., 2011; Anasori et al., 2017). These materials have attracted much attention due to their physical properties such as structural tunability, metal properties, carrier migration anisotropy and good optical properties. Lin et al. synthesized a new type of ultra-thin 2D niobium carbide (Nb_2_C) MXene by a two-step liquid stripping method (Lin et al., 2017a). The synthesis and layering of ultra-thin 2D Nb_2_C (MXene) were attained by HF etching combined with tetrapropylammonium hydroxide intercalation (TPAOH) (Figure 4G). The absorption spectrum of Nb_2_C NSs shows a board absorption band in both NIR-I and NIR-II regions (Figure 4H), and the extinction coefficients at 808 and 1,064 nm also reached 37.6 L g^−1^ cm^−1^ and 35.4 L g^−1^ cm^−1^, respectively. It can be seen that Nb_2_C NSs have strong light absorption capacity in NIR-II and NIR-II. By measuring the temperature changes of Nb_2_C solution under different power density laser irradiation, it was found that the temperature is positively related with the laser power density. The solution under the laser with the maximum power density (1.5 W cm^−2^) in the experiment increased to 60°C within 5 min, indicating that Nb_2_C can quickly and efficiently convert NIR light into heat energy **(** Figure 4I **)**. In addition, good photothermal stability was confirmed by repeatedly excitation at 808 and 1,064 nm lasers for 5 times. Finally, after measuring the heat transfer time constant and the maximum steady state temperature, it was determined that the photothermal conversion efficiency of Nb_2_C NSs at 808 and 1,064 nm reached 36.5 and 46.65%, respectively, which proves the potential of Nb_2_C NSs as a photothermal reagent (Lin et al., 2020b). Han et al. proposed to construct “therapeutic mesopores” on the surface of 2D Nb_2_C MXenes. CTAC was self-assembled onto Nb_2_C MXenes by electrostatic interaction, then organosilane source is added to the reaction system to form a mesoporous silica shell on the surface of 2D Nb_2_C MXenes (CTAC@Nb_2_C-MSN). The prepared CTAC@Nb_2_C-MSN shows strong light absorption in the NIR biological window (Han et al., 2018). By comparing the temperature changes of CTAC@Nb_2_C-MSN solutions with different concentrations under the irradiation of 1,064 nm laser, it is found that the temperature of the solution after photothermal conversion rises with the increase of the solution concentration. Furthermore, the temperature after photothermal conversion also rises with the increase of the power density of the 1,064 nm laser. Liu et al. (2020a) used phenol as the precursor, through one-step solvothermal treatment, adjusted the decomposition of hydrogen peroxide under a 9T magnetic field to synthesize 9T-GQDs, and prepared 0T-GQDs without introducing a strong external magnetic field. 9T-GQDS dispersions of different concentrations were irradiated with 1,064 nm laser for 300 s, and it was found that the temperature of photothermal conversion was proportional to the concentration of the solution. The temperature change of 9T-GQDs after NIR-II laser irradiation for 780 s was also measured, the maximum temperature of the solution after photothermal conversion reached nearly 50°C, showing excellent photothermal conversion ability. In addition, many researchers have studied the photothermal effects of 2D materials. Tang et al. (2019b) synthesized 2D core-shell nanocomposites (Ti_3_C_2_@Au) by seed growth method, and the light absorption capacity and photothermal conversion efficiency were improved by the thiol modification of the surface. Wang et al. synthesized ultra-thin polypyrrole nanosheets (PPy) by a space-constrained method (Yanai et al., 2008), which can change the electronic structure and optical properties by doping PPy. The doped PPy not only showed strong light absorption ability in UV-vis-NIR spectrum, but also improved the photothermal conversion rate (Wang et al., 2018a). Xu et al. (2019) prepared a hydrogel drug carrier based on 2D tungsten nitride nanosheets, which shows good photothermal sensitivity and high photothermal conversion efficiency under NIR-II laser irradiation. Xu et al. (2020a) summarized the preparation methods, photothermal conversion mechanisms and related applications of several 2D materials. Dong et al. (2020) described the latest developments of MXenes materials in terms of structure and properties, and summarized the unique advantages of MXenes in PTT. Wang et al. summarized the main applications of 2D nanocomposites in biomedicine due to their excellent photothermal properties (Wang and Cheng, 2019). 2D materials have completely different energy band structures and optical properties, and these characteristics endow them with great potential for future development. At present, the research on the preparation, characterization and modification of 2D materials has made great progress, and the unique characteristics of 2D materials have provided more possibilities for the development and progress of various fields such as biomedicine, communications, and electrochemistry.

### Metal Sulfide Oxide Materials

**FIGURE 5:**
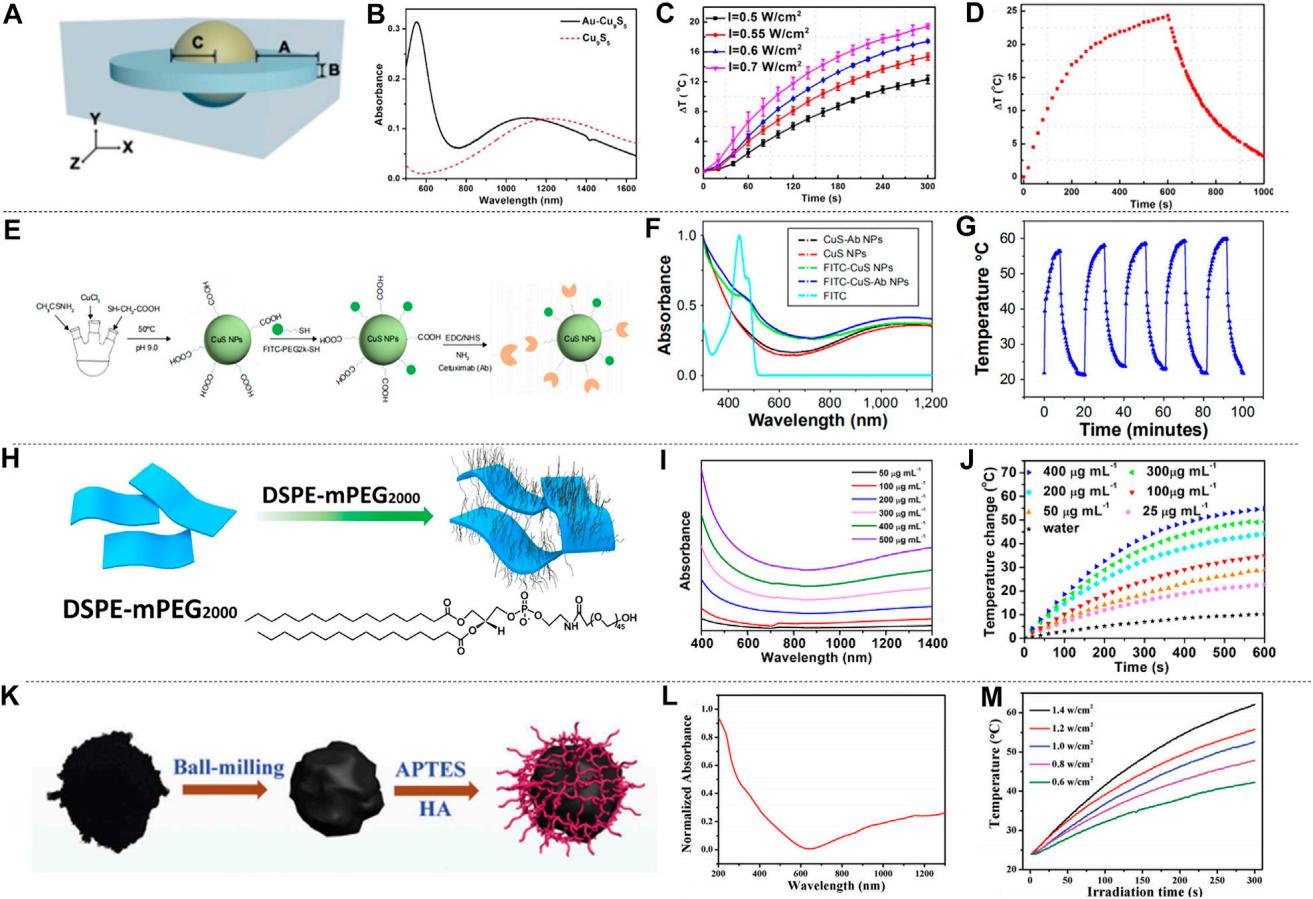
Photothermal characteristics of metal sulfur oxides. **(A)** DDA simulation structure diagram of Au-Cu_9_S_5_ NPs. **(B)** The absorption spectrum of Au-Cu_9_S_5_ NPs. **(C)** Temperature characteristic curves of Au-Cu_9_S_5_ NPs solutions excited by 1,064 nm with different power densities. **(D)** Temperature change graph of Au-Cu_9_S_5_ NPs solution excited by 1,064 nm laser (reproduced from (Ding et al., 2014) with permission from American Chemical Society). **(E)** Synthesis of CuS-Ab NPs. **(F)** Absorption spectrum of CuS-Ab NPs. **(G)** The temperature change curve of the CuS-Ab NPs solution irradiated by a 1,064 nm laser for five cycles (reproduced from (Li et al., 2018) with permission from Dove Medical Press LTD.). **(H)** Schematic diagram of DSPE-mPEG wrapped TONW NRs. **(I)** Absorption spectra of different concentrations of PEG-TONW NRs solutions. **(J)** The temperature rise curve of PEG-TONW NRs solution with different concentrations irradiated by a 1,064 nm laser (reproduced from (Cheng et al., 2019) with permission from American Chemical Society). **(K)** Titanium dioxide synthesis diagram. **(L)** Absorption spectrum of Ti_2_O_3_ NPs. **(M)** Temperature-time graphs of Ti_2_O_3_ NPs irradiated by 1,064 nm lasers with different power densities (reproduced from (Zeng et al., 2018) with permission from Royal Society of Chemistry).

Metal sulfide and oxide materials have attracted wide attention because of their special properties such as free electron transfer characteristics, magnetism (Gao et al., 2017), structure, and photothermal stability (Dahoumane et al., 2016; Ge et al., 2016; Vena et al., 2016; Guo et al., 2020). The unique vacancy structure of copper sulfide, and the LSPR caused by the conduction electron resonance oscillation provide possibility for the study of PTT in NIR-II. Ding et al. (2014) synthesized dual plasma gold copper sulfide nanomaterials (Au-Cu_9_S_5_ NPs) by seed-mediated growth combining Au and Cu_9_S_5_, and simulated the Au-Cu_9_S_5_ nanostructure with discrete dipole approximation (DDA). The structure of Au-Cu_9_S_5_ is shown in Figure 5A, and Au-Cu_9_S_5_ NPs have enhanced light absorption and unique optical properties. Both the absorption spectra of Au-Cu_9_S_5_ and Cu_9_S_5_ were show in Figure 5B. Au-Cu_9_S_5_ NPs have two absorption peaks in the visible and NIR region, the peak at 550 nm is due to the red-shift of the absorption peak of Au NPs, and the absorption peak at 1,100 nm is the absorption peak of Cu_9_S_5_ NPs. When 1,064 nm lasers with different power densities were used to irradiate Au-Cu_9_S_5_ NPs solution (Figure 5C), the rising trend of the temperature was subsequently increased. When the Au-Cu_9_S_5_ NPs solution was irradiated for 300 s with a power density of 1,064 nm laser as low as 0.5 W cm^−2^, the temperature increased by 12.3°C, which was much higher than the temperature rise required for tumor PTT. The Au-Cu_9_S_5_ NPs solution was irradiated with a 1,064 nm laser for 10 min, after natural cooling for more than 1,000 s, the temperature difference curve of Au-Cu_9_S_5_ NPs is shown in Figure 5D. During the laser irradiation, the temperature of the Au-Cu_9_S_5_ NPs solution rose, removed the light source and the temperature naturally dropped. The phenomenon proved that Au-Cu_9_S_5_ NPs have excellent photothermal transduction characteristics. Wang et al. (2020d) synthesized the CuS-Au heterostructure by anion and cation synthesis method. The CuS-Au heterostructure has strong LSPR, photostability and photothermal conversion effects, proving that the CuS-Au heterostructure is an efficient and stable photothermal agent. Wu et al. (2015) proposed to embed Fe_3_O_4_ NPs synthesized by thermal decomposition into hollow and porous CuS NPs to produce rattle-type Fe_3_O_4_@CuS nanoparticles (Fe_3_O_4_@CuS NPs). The absorption spectrum of Fe_3_O_4_@CuS NPs is in the range of 1,000–1,350 nm. The photothermal ablation method was used to evaluate the photothermal conversion performance of Fe_3_O_4_@CuS NPs and the results showed that the Fe_3_O_4_@CuS NPs solution has better performance under 1,064 nm laser irradiation than 808 nm. In addition, copper sulfide can also be combined with some non-metallic substances to increase the accumulation of copper sulfide in tumor cells, which is also an important research direction. Cetuximab is an antibody against epidermal growth factor receptor (EGFR) over-expressed on the cell membrane. Li et al. (2018) proposed to modify copper sulfide (CuS-Ab NPs) with cetuximab to improve the accumulation of CuS nanoparticles (CuS NPs) in tumor cells, as shown in Figure 5E. CuS-Ab NPs have an absorption peak at 1,065 nm (Figure 5F). The CuS-Ab NPs solution was irradiated with 1,064 nm laser for five cycles, and it was found that the solution temperature rose to the highest point under laser irradiation, after the light source was removed, the solution temperature naturally decreased (Figure 5G). The solution temperature trends of the five cycles are basically the same, indicating that the CuS-Ab NPs have strong photostability. Zhou et al. (2015) used a one-pot method to introduce the tumor-specific targeting ligand FA into CuS NPs (FA-CuS NPs). FA-CuS NPs not only have high photostability and photothermal conversion characteristics, but also have the ability of targeting cancer cells, which is helpful for PTT. Because of the high X-ray attenuation coefficient of bismuth, it can be used as a radio-sensitizer, and the researchers combined radiotherapy and PTT to study copper bismuth sulfide. Du et al. (2017) established a D-α-tocopherol polyethylene glycol 1,000 succinate functionalized Cu_3_BiS_3_ nanocrystal (TPGS-Cu_3_BiS_3_ NCs) system. It uses the characteristics of bismuth to concentrate radiation energy on tumor cells and improve treatment efficiency. Li et al. (2017a) prepared PEGylated Cu_3_BiS_3_ NRs nanorods. The nanorods show an absorption peak at 1,065 nm, have strong NIR absorption and a large bismuth X-ray attenuation coefficient, realizing the combination of radiotherapy and PTT. In addition to the research on copper group sulphides, scholars have also explored some other sulfides. Li et al. (2021) studied the diversified nanoplatform Ag_2_S@MSN-TGF, and Han et al. (2021) synthesized water soluble silver sulfide nanoparticles with optimal size (Ag_2_S NPs). They all used the fluorescence effect of silver sulfide and the photothermal conversion characteristics in NIR-II to track the PTT process. Lei et al. (2019) used the two-phase method to synthesize nickel sulfide nanoparticles (Ni_9_S_8_ NPs) for the first time. Under 1,064 nm laser irradiation, its temperature increased with the increase of the concentration of the solution, which proved that Ni_9_S_8_ NPs had good photothermal treatment potential.

Metal oxides also attracted wide attention. Ammonium-tungsten-bronze ((NH_4_)xWO_3_) nanomaterials have the characteristics of low cost, wide light absorption area, high extinction coefficient, etc., and have great research potential in PTT of NIR-II. Cheng et al. (2019) proposed the two-dimensional ultrathin tellurium oxide/(NH_4_)xWO_3_ bronze (TeO_2_/(NH_4_)xWO_3_) nanoribbons (TONW NRs). Due to the LSPR of free electrons, the nanoribbons exhibited strong near-infrared absorption. Compared with ammonium tungsten bronze nanomaterials, TOWN NRs had an electronic transition between the arc electron pairs on W^6+^ and Te atoms, which further improved the NIR-II absorption of (NH_4_)xWO_3_. In order to improve the solubility of TONW NRs, the nanobelt was wrapped in DSPE-PEG (1,2-distearoyl-sn-glycerol-3-phosphoethanolamine-n-[methoxy (polyethylene glycol)-2000) to form PEG-TONW NRs (Figure 5H). and the absorption spectra of different concentrations of PEG-TONW NRs solutions were shown in Figure 5I. PEG-TONW NRs show great PTT potential in NIR-II. Different concentrations of PEG-TONW NRs solution were irradiated with 1,064 nm laser, and the temperature change of the solution was shown in Figure 5J. The temperature of the PEG-TONW NRs solution rose faster over time, and the increment was correlated with the concentration of PEG-TONW NRs. Guo et al. (2015) prepared (NH_4_)xWO_3_ `nanocubes by high-temperature solvothermal method. Due to the strong LSPR of the (NH_4_)xWO_3_ nanocube, the dried (NH_4_)xWO_3_ has a strong absorption at 780–2,500 nm, indicating its potential in photothermal treatment. In addition to low cost and photothermal stability, some metal oxides can also be targeted to tumor cells, which can improve the effect of PTT and has been widely studied. Zeng et al. (2018a) prepared hyaluronic acid (HA) modified titanium oxide nanoparticles (Ti_2_O_3_@HA NPs) by ball milling method, as shown in Figure 5K. The absorption peak of Ti_2_O_3_@HA NPs is between 1,000–1700 nm (Figure 5L). Ti_2_O_3_@HA NPs solution was irradiated with 1,064 nm lasers with different power densities, the temperature of the solution was positively correlated with the power density and showed a time dependence (Figure 5M), which indicated that Ti_2_O_3_@HA NPs have good photothermal conversion efficiency and PTT potential. Liu et al. (2020b) synthesized chitosan (CS) coated RuO_2_ NPs (CS-RuO_2_ NPs) with a one-pot method, which has unique potential in low-temperature PTT. Guo et al. (2020) synthesized MoO_2_ nano-aggregates by hydrothermal synthesis. It has strong cell penetration at 1,064 nm. The oxidative degradation of MoO_2_ makes them available for PTT.

The above research proved that metal sulfide oxides have great application potential on the PTT in NIR-II region. The combination of sulfides and oxides with targeting antibodies and mixed synthesis with other materials provide a good idea and direction for future research. Reducing the synthesis temperature and simplifying the synthesis method to make the material have its own unique properties, or making the materials have longer absorption wavelength to achieve a greater cell penetration are all worth exploring.

### Polymers

A polymer is usually organic and composed of structural units called monomers. The structural units are repeatedly arranged into sub-chains, which can reach hundreds of nanometers. Some polymers have been proved to have good photothermal conversion efficiency and photothermal stability in NIR-II.

**FIGURE 6:**
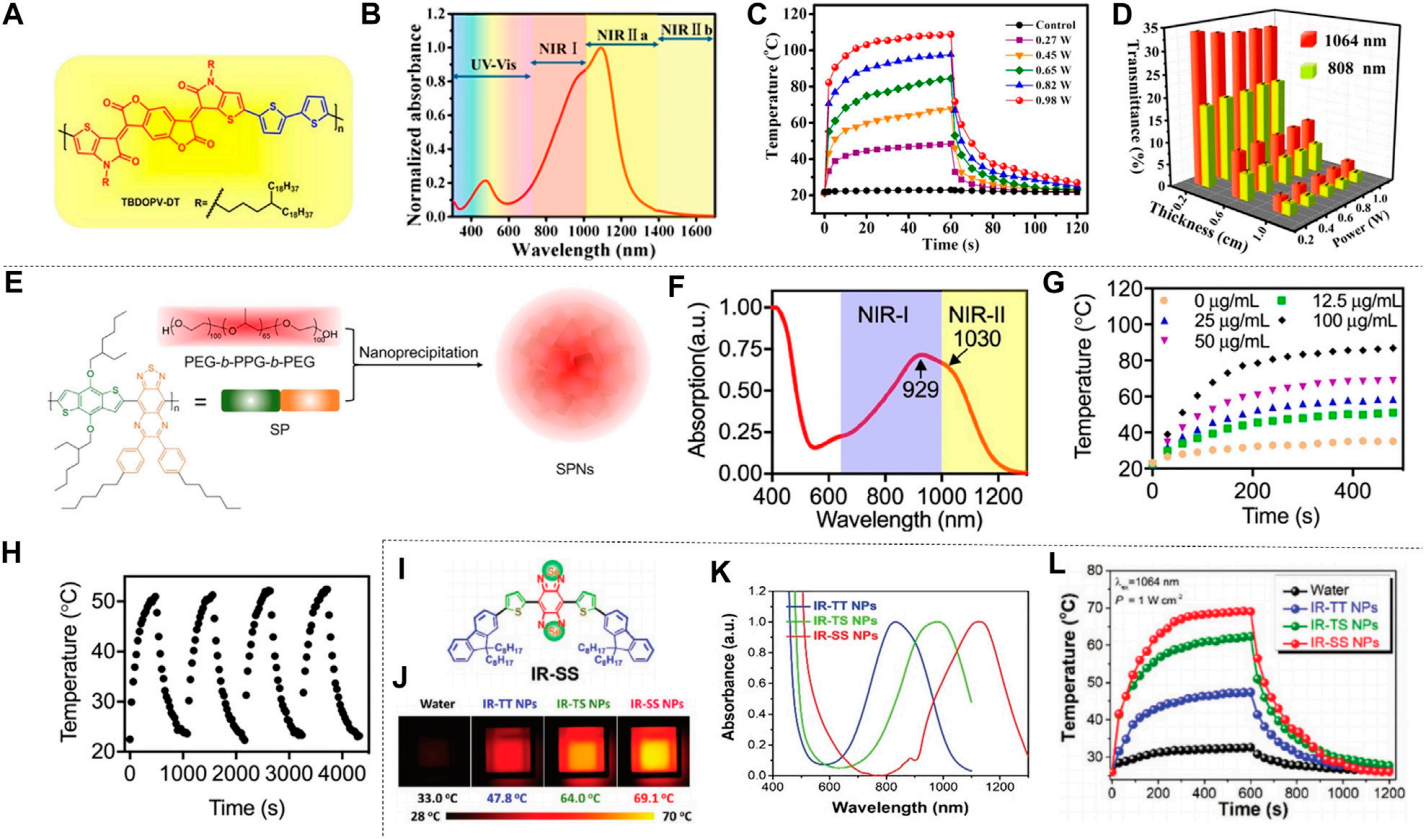
Photothermal properties of polymer nanomaterials. **(A)** TBDOPV-DT’s chemical structure red markings for the receptor part, and blue for the donor part. **(B)** Standardized UV-vis-NIR spectrum of TBDOPV-DT membranes. The spectral regions are segmented into Vis (300–750 nm), NIR-I (750–1,000 nm), NIR-Ⅱa (1,000–1,400 nm), and NIR-Ⅱb (1,400–1700 nm). **(C)** At different power levels, the TBDOPV-DT film’s surface temperature is increased or decreased by lighting. Use the glass substrate with an uncoated TBDOPV-DT film as a control sample. **(D)** Transmission comparison between 1,064 nm and 808 nm laser at different pig skin thicknesses (0.2, 0.6 and 0.0 cm) and different powers (0.27, 0.45, 0.45, 0.65 and 0.82 W) (reproduced from (Cao et al., 2016) with permission from American Chemical Society). **(E)** Description of the SPNs of SP and PEG-b-PPG-b-PEG was prepared by nanoprecipitation. **(F)** The SPNs absorption spectra measured in an aqueous medium. **(G)** SPNs’photothermal effect. (Laser wavelength, 1,064 nm; strength, 1 W cm^−2^). **(H)** SPNs’ speriodic optical heating. Concentration is 12.5 μg ml^−1^ (reproduced from (Wen, at al., 2020) with permission from American Chemical Societyl). **(I)** Structure of IR-SS. **(J)** The infrared imaging of three NPs dispersion serves as a function of irradiation time. **(K)** for standardized absorption spectroscopy of three NPs aqueous solutions. **(L)** A photothermal heating curve of 10 min performed at 1064 nm irradiation (1 W cm^−2^) (reproduced from (Li et al., 2020) with permission from Wiley-Blackwell).

The organic conjugated polymer is an ideal material for NIR absorption, which can promote the effective NIR-II photothermal conversion by reducing its absorption range in the NIR-II. Cao et al. proposed an effective strategy for increasing π conjugate length by designing a quinoline stabilized donor receptor (D-A) polymer. A D-A conjugated polymer (TBDOPV-DT) with a narrow band gap was synthesized using p-phenylene vinylene as the acceptor and 2,2' bithiophene as the donor (Figure 6A). TBDOPV-DT is a NIR-II photothermal material whose maximum absorption peak (1,093 nm) falls exactly into the NIR-II (Figure 6B). Therefore, the polymer TBDOPV-DT shows high photothermal conversion efficiency under the irradiation of 1,064 nm wavelength laser. The polymer film’s surface temperature increased significantly under the irradiation of 1,064 nm laser, which proved good photothermal conversion of TBDOPV-DT in the NIR-II (Figure 6C). With increasing laser power, the surface temperature of the polymer film increased gradually and was almost in proportion to the laser power. The TBDOPV-DT film’s photostability was assessed by repeating light irradiation at 0.45 W cm^−2^. After 10 heating and cooling cycles, the surface temperature remained the same during the unchanged irradiation time, which further verified its excellent thermal stability. What’s more, the material is highly soluble in organic solvents, and can be easily assembled into NPs by emulsion method. These NPs are relatively uniform and their size can be adjusted by the emulsification rate. To verify the deep tissue penetration capacity of NIR-II light, the transmissivity of 1,064 nm laser and 808 nm laser were compared. Experiment showed that the penetrating power of the 1,064 nm laser is approximately twice that of the 808 nm laser (Figure 6D). Cao et al. (2016) therefore developed a narrow-band-gap conjugated polymer TBDOPV-DT with ability to efficiently absorb NIR-II light for efficient photothermal conversion. Similarly, Guo et al. (2018) used D-A structure conjugated polymers with excellent NIR-II absorption to prepare NPs by chemical modification of active targeting ligand Cyclo (Arg-Gly-Asp-D-Phe-Lys (MPA)) (C-RGD) after simple nano-precipitation. This material also showed ideal photothermal properties. Wen et al. obtained semiconducting polymer nanoparticles (SPNs) using strong electron donor and acceptors to narrow the band gap, which also has effective optical response in NIR-II (Figure 6E). The absorption spectrum of SPNs in water dispersion is shown in Figure 6F, the absorption peak of NIR-I window is 929 nm, and that of NIR-II window is 1,030 nm. Such a wide absorption band allows SPNs to perform photothermal conversion in two NIR Windows. In order to assess the photothermal effect of NIR-II, a 1,064 nm pulsed laser was applied to illuminate the water solution of SPNs with different concentrations for 8 min. The result indicated that the temperature was positively correlated with the irradiation time and the concentration of SPNs (Figure 6G). In particular, the photothermal temperature of 100 μg/ml SPNs can reach 85.6°C after 8 min excitation. The photothermal stability was studied by analyzing the SPNs dispersion’s “hot and cold” period (Figure 6H). The result showed that the maximum temperature was almost constant in the four cycles of the laser (1,064 nm, 1.0 W cm^−2^) on/off. The alternate electron donor-acceptor structure exhibited ideal photothermal properties (Wen et al., 2020b). Sun et al. (2019a) synthesized semiconducting polymers using a tripolymer method with one electron acceptor unit and two electron-doping units. The absorption characteristics can be modulated by adjusting the molar ratio of the three monomers. Different from the above methods, π-conjugated small molecules can perfectly regulate the optical absorption from NIR-I to NIR-II by molecular engineering in which individual atoms are replaced. Using this technique, Li et al. developed a conjugated oligomer IR-SS molecule with an absorption peak of greater than 1,000 nm (Figure 6I). Li et al. used twisted fluorene as a donor and benzo [1, 2-C: 4, 5-C'] bis ([1, 2, 5] thia-diazole) (BBT) as a strong receptor to construct NIR absorbent compounds (IR-TT). When one or two S atoms were replaced by selenium (Se) atom, IR-TS and IR-SS were prepared. The absorption peaks of IR-TT, IR-TS and IR-SS are 830 nm, 975 and 1,120 nm, respectively (Figure 6K). The photothermal conversion ability of IR-TT, IR-TS and IR-SS NPs water solutions (100 μg ml^−1^) under the irradiation of NIR-II light was studied using an infrared camera. As shown in Figure 6L, after 5 min all NPs’ temperature increased rapidly at 1,064 nm laser irradiation (1 W cm^−2^). The temperature of IR-TT, IR-TS and IR-SS NPs aqueous solutions severally reached 47.8 °C, 64.0°C and 69.1 °C. The corresponding infrared images are shown in Figure 6J. After the thermal cycles of the five laser switches, the photothermal properties of the IR-SS NPs did not change significantly, which proved that it has high photothermal stability. In conclusion, Li et al. (2020a) precisely tuned a small molecule at the atomic level to transform it from the NIR-I to the NIR-II region for optical absorption using molecular engineering, and the IR-SS NPs have high photothermal energy conversion efficiency at 1,060 nm.

Jiang et al. prepared semiconductor copolymer nanoparticles (SPNI) with bimodal absorption of NIR-I and NIR-II, which have two different fragments that severally absorb the light of NIR-I and NIR-II. The photothermal conversion efficiency of SPNI-II at 1,064 nm is usually better than that of other inorganic nanomaterials. The maximum temperature of the SPNI-II solution under NIR-II was approximately 1.8 times the respective maximum permissible exposure limit under NIR-I radiation. This result showed that NIR-II is better to NIR-I in photothermal heating of SPNI-II. In addition, SPNI-II also showed outstanding photothermal stability. This material represents the first organic photothermal nano-agent with peak absorption in the NIR-I and NIR-II regions (Jiang et al., 2018a). Cao et al. (2018b) also prepared another semiconducting polymer, PBTPBF-BT nanomaterials, based on the thieno-isoindigo derivative-based semiconducting polymer, which has strong photothermal conversion efficiency and excellent photothermal stability.

### Other Materials

Other materials that have good photothermal conversion efficiency such as carbon nanomaterials and NIR-II dyes have been being developed for PTT. Xu et al. designed and synthesized hollow carbon nanosphere modified with polyethylene glycol-graft-polyethylenimine (HPP). The HPP exhibited some exceptional properties as a photothermal agent such as uniform core-shell structure, good biocompatibility and excellent heat conversion efficiency. Upon NIR-II laser irradiation, the intracellular HPP showed an excellent photothermal conversion efficiency of 45.1% and high activity towards cancer cell killing. In addition, the extended biomedical application as drug carrier was also achieved utilizing the large internal cavity of HPP. All the results promised the HPP as a great potential in NIR-II laser-activated cancer photothermal therapy (Xu et al., 2021). Yang et al. fabricated a novel nanocarrier composed of biodegradable charged polyester vectors (BCPVs) and graphene quantum dots (GQDs) for pancreatic cancer therapy applications. The nanocarrier was utilized to co-load doxorubicin (DOX) and small interfering ribonucleic acid (siRNA). The GQD/DOX/BCPV/siRNA nanocomplexes were triggered by light to exert photothermal treatment and release the loaded drugs simultaneously (Yang et al., 2019). Cui et al. (2021) constructed lipid-bilayer-coated reduced graphene oxide (rGO) modified with mesoporous-silica nanosheets as a NIR light-activated drug carrier and photothermal agents. Enriched graphitic N doped carbon dots (NIR–II–CDs) showed an ultrahigh photothermal conversion efficiency of 81.3% when the graphitic N doping level was 4.3%. The carbon dots can rapidly increase the tumor temperature from 35.8 to 48.6°C within 5 min upon the 1,064 nm laser irradiation, in addition, it also can work as drug carrier and release the chemotherapeutics under NIR- II laser to facilitate the comprehensive curative effect (Geng et al., 2020). Other carbon nanomaterials such as carbon dots (CDs), carbon nanotubes, Graphene quantum dots (GQDs), carbon fiber, carbon nano-onion clusters and carbon-based nanocomposite like degradable carbon−silica nanocomposite (CSN) with high photothermal conversion efficiency were developed for cancer photothermal treatment, and exhibited promising prospect of carbon materials in NIR-based PTT (Thakur et al., 2017; Geng et al., 2018; Gong et al., 2018; Permatasari et al., 2018; Li et al., 2019b; Lu et al., 2019; Sun et al., 2019b; Wang et al., 2020b).

NIR-II dyes utilized for PTT not only can perform photothermal ablation of tumors but also can guide the treat by taking the advantage of NIR imaging with deep tissue penetration. Li et al. developed an ingenious macromolecular fluorophore (PF) by conjugating a small-molecule NIR-II fluorophore (Flav7) with an amphiphilic polypeptide. The PF nanoparticles showed satisfactory prominent photothermal conversion efficiency (42.3%) upon 808 nm excitation, excellent photothermal stability and good biocompatibility. *In vivo* studies revealed that the PF nanoparticles realized excellent photothermal ablation effect on 4T-1 tumors and the process can be traced by NIR fluorescence imaging (Li et al., 2019a). Yao et al. synthesized a NIR-II dye SQ1 by introducing malonitrile into a D−A−D NIR-I structure composed of ethyl-grafted 1,8-naphtholactam as donor units and square acid as acceptor unit. The photothermal conversion efficiencies of SQ1 nanoprobe was 25.6%, and temperatures of tumors in mice bearing MDA-MB-231 xenograft injected with SQ1 nanoprobe increased rapidly from 33.2 to 59.1°C (Yao et al., 2020). Zeng et al. (2018b) conjugated water-soluble and biocompatible small-molecule NIR-II dye H2a-4T with fetal bovine serum (FBS) and Cetuximab proteins to enhance the tumor targeting and achieved good PTT effect on colorectal cancer. Assorted dyes with NIR-II fluorescence were adopted and developed for use in PTT with good photothermal effect, and showed promising perspective of NIR-II dyes utilized for imaging-guided PTT (Wang et al., 2019; Zhou et al., 2020a; Xu et al., 2020b; Chen et al., 2020; Sun et al., 2020).

Gold nanomaterials, two-dimensional materials, metal sulfide oxide materials, polymers, carbon nanomaterials and NIR-II dyes are all good candidates for PTT utilization and being developed progressively. High photothermal conversion efficiency and favorable biocompatibility will facilitate the clinical translation, and the NIR-II PTT will become available soon for cancer treatment in clinic. Some representative materials mentioned above are shown in Table 1.

**TABLE 1:** Summary of the properties and applications of representative NIR-Ⅱ PTT materials discussed in this review (Ex, excitation wavelength (nm); PCE, photothermal conversion efficiency (%); ΔT, temperature increased (°C); NA, not applicable).

Category	Name	Ex	PCE	Application	ΔT	Reference
Gold nanomaterials	Au_3_Cu TPNCs	808, 1,064	39.45, 75.27	NIH3T3 and KB cells, KB tumor-bearing mice	19.8 (1,064)	Wang, et al. (2018c)
	AuNPL@TiO_2_	808, 1,064	NA, 42.05	HeLa cells, HeLa tumor-bearing mice	＞ 40 (1,064, solution)	Gao et al. (2019)
Two-dimensional materials	NIR-II-CD/BP	808, 1,064	77.3, 61.4	HeLa cells, 4T1 tumor-bearing mice	15.3 (1,064)	Geng et al. (2019)
	MoS_2_	1,064	44.3 (1T-MoS_2_)	A549 and HeLa cells, A549 tumor-bearing mice	28.3 (1T-MoS_2_))	Zhou et al. (2020b)
	MXene (Nb_2_C−PVP)	808, 1,064	36.4, 45.65	4T1 and U87 cells, 4T1 tumor-bearing mice	31, 35	Lin et al. (2017a)
Metal sulfide and oxide materials	CuS-Au	1,064	36.5	4T1 tumor-bearing mice	17	Han et al. (2021)
	PEG-TONW NRs	1,064	43.6	MCF-7 cells and A549 cells, MCF-7 tumors-bearing mice	28	Cheng et al. (2019)
	CS-RuO2NPs	1,064	52.5	MCF-7 cells, MCF-7 tumor-bearing mice	12	Liu et al. (2020b)
Polymers	P1RGD NPs	1,064	30.1	U87 cells, U87 tumor-bearing mice	16	Guo et al. (2018)
	SPN	1,064	21.3	U87 cells, U87 tumor-bearing mice	11	Wen et al. (2020b)
	IR-SS	1,064	77	4T1 tumor-bearing mice	19	Li et al. (2020a)
	NP_PBTPBF-BT_	1,064	66.4	MDA-MB-231 cells, MDA-MB-231 tumor-bearing mice	35	Cao et al. (2018b)
	SPN_I-II_	1,064	43.4	4T1 tumor-bearing mice	22.7	Jiang et al. (2018a)
Carbon	HPP	1,064	45.1	4T1 cells, 4T1 tumor-bearing mice	15	Xu et al. (2021)
Nanomaterials	N-doped NIR-II-CD	808, 1,064	85.7, 81.3	HeLa cells, 4T1 tumor-bearing mice	12.8 (1,064)	Geng et al. (2020)
NIR-II dyes	PF	808	42.3	4T1 and HepG2 cells, 4T1 tumor-bearing mice	16	Li et al. (2019a)
	IR1048-MZ	980	20.2	A549 tumor-bearing mice	28	Meng et al. (2018)

## Photothermal Treatment

PTT in NIR-II window can kill cancer cells in the organism with photothermal ablation. It has the advantages of easy operation, non-invasion, deep tissue penetration, and high temporal and spatial resolution (Lyu et al., 2019). Its use in cancer treatment has promising prospects and significance in the field of medical research.

Cancer has always been one of the major threats to human life (Jemal et al., 2011). Traditional treatment methods such as surgery, chemotherapy, radiotherapy, and other comprehensive therapies are used in clinical treatment for different cancers (Bray et al., 2012). However, the side effects of these traditional cancer treatments usually cause great harm to the human body. Therefore, it is necessary to develop efficient and non-invasive cancer treatment methods to settle these problems. PTT has brought great hope for this in terms of temporal and spatial addressability, minimal invasiveness, and high therapeutic effect, which has received extensive attention in recent years (Lu et al., 2015; Zhang et al., 2017; Wei et al., 2018; Yang et al., 2018; Zhang et al., 2018). This method kills cancer cells by converting light into heat (Melamed et al., 2015). In the past few decades, the field of photothermal nano-preparations for biomedical applications has made great progress, and a variety of different materials have been developed for the photothermal treatment of various cancers.

### Breast Cancer

In recent years, breast cancer has received widespread attention because it has the characteristics of high prevalence, ambiguous etiology, and difficult treatment. Moreover, due to the relatively high incidence and mortality, breast cancer is also known as the “invisible killer” of women. Breast cancer cell lines mainly include 4T1, MCF-7 (Cheng et al., 2019; Liu et al., 2020b; Zhang et al., 2020d), and MDA-MB-231 (Cao et al., 2018b; Wang et al., 2020c) in recent researches. Among them, 4T1 breast cancer cell lines are the most commonly used in experimental studies.

**FIGURE 7:**
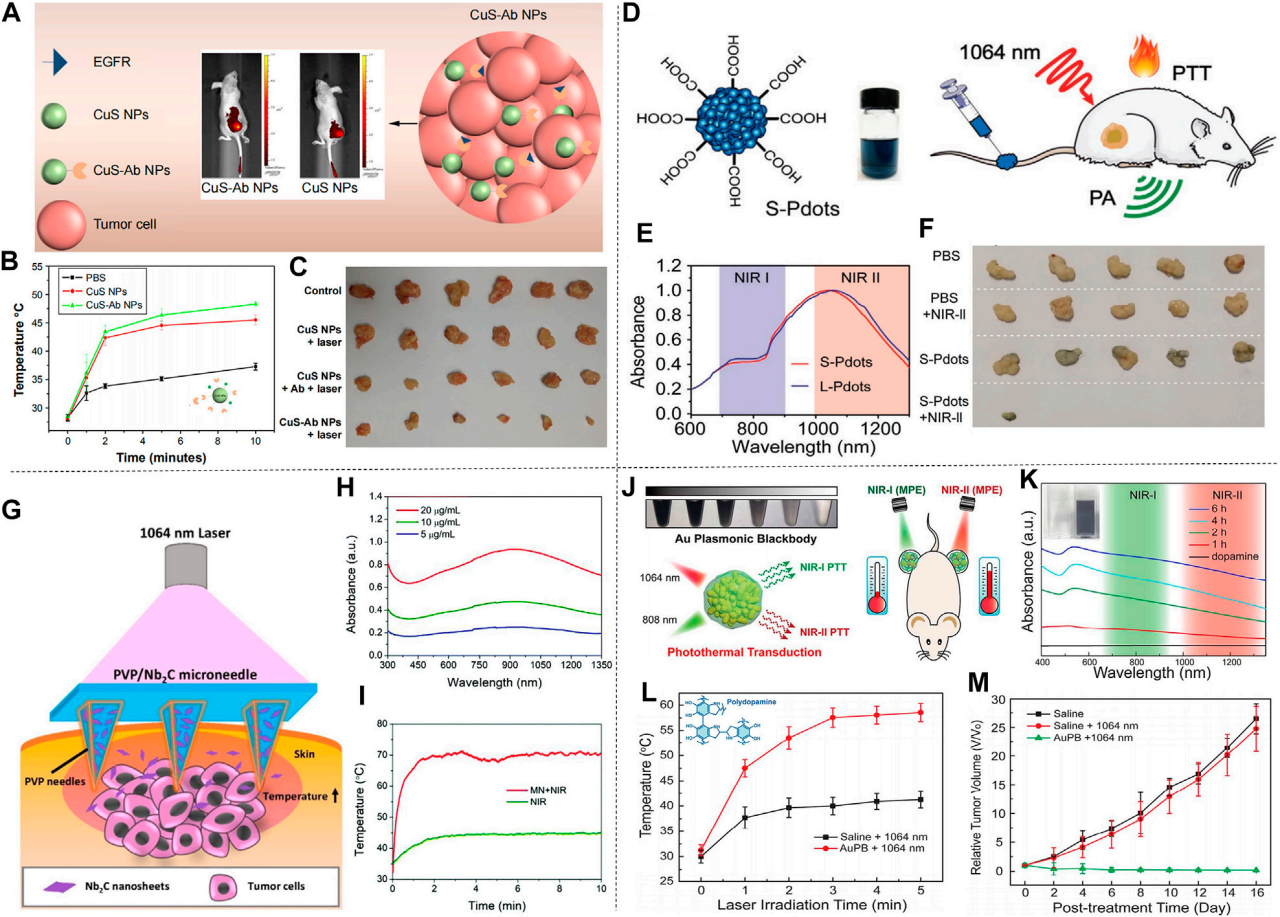
Treatment effect of mice with 4T1 breast cancer cells. **(A)** Illustration of CuS-Ab NPs’ target tumor. **(B)** Tumor temperature change curve in mice after treatment with CuS NPs and CuS-Ab NPs. **(C)** Photos of tumors during treatment in four groups of mice (reproduced from (Li et al., 2018) with permission from Dove Medical Press Ltd.). **(D)** Molecular structure diagram of DPP-BTzTD Pdots and its use for PTT. **(E)** NIR absorption spectra of S-Pdots and L-Pdots. **(F)** Tumors cut from mice in different groups (reproduced from (Men et al., 2020) with permission from Wiley-VCH Verlag). **(G)** Diagram of 2D MXene-based therapeutic microneedle patch used for superficial tumor phototherapeutics. **(H)** The UV-vis-NIR absorption spectra of MN in different concentration. **(I)** Tumor temperature change curve in NIR group and MN + laser group (reproduced from (Lin et al., 2020) with permission from Royal Society of Chemistry). **(J)** Schematic of AuPBs under NIR laser. **(K)** UV-vis-NIR absorption spectra of dopamine and AuPBs in different condition. **(L)** Temperature change curve of tumor in two groups. **(M)** Relative Tumor growth curve of three group after treatment (reproduced from (Zhou et al., 2018) with permission from American Chemical Society).

Li et al. proposed CuS nanoparticles modified with Ab (CuS-Ab NPs) (Li et al., 2018). The introduction of Ab anti-angiogenic antibody increased the accumulation of CuS NPs in tumors, which inhibited the formation of blood vessels to further achieve the inhibition of cancer cell growth and obtained a better therapeutic effect (Figure 7A). CuS-Ab NPs have a maximum absorbance at 1,065 nm, indicating that these NPs have potential photothermal properties in NIR-II. In addition, experiments showed that CuS-Ab NPs have little damage to biological tissues and have amazing photothermal stability, which benefits its further development. Based on the *in vitro* photothermal characteristics of CuS-Ab NPs, the *in vivo* anti-tumor efficacy was explored. 24 mice with 4T1 tumors were divided into four groups: 1) control; 2) CuS NPs + laser; 3) CuS NPs + Ab + laser; 4) CuS-Ab NPs + laser. After the experiment started, the temperature of the fourth group rises the fastest, as show in Figure 7B. The body weight and tumor volume of the mice were measured every day, and it was found that CuS-Ab NPs could inhibit the tumor growth in mice under 1,064 nm laser irradiation. The tumor image demonstrates clearly that as the treatment time increased, the tumor significantly shrunk (Figure 7C), while the other three groups had no significant changes in tumor volume, indicating that the CuS-Ab NPs + laser group can effectively inhibit the growth of tumor and realize photothermal treatment. During the whole treatment process, the body weight of the mice did not change significantly, indicating that CuS-Ab NPs have little biological toxicity. Metal sulfide oxides are widely used as photothermal agents. In addition to CuS (Wang et al., 2020d), there are some other nanomaterials commonly used for PTT of cancer, such as Ag_2_S (Li et al., 2021), Cu_3_BiS_3_ (Li et al., 2017a), (NH_4_)WO_3_ (Guo et al., 2015), etc. Men et al. (2020) constructed a new type of ultrasmall multifunctional polymer quantum dots (Pdots) (Figure 7D), which was obtained by self-assembled polymer via hydrophilic and hydrophobic interaction using modified nanoprecipitation method for the synthesized DPP-BTzTD. The particle size of Pdots was 4 nm, smaller than the traditional particle size of 10–200 nm, attempting to solve the problem of difficult elimination from the body. It can be seen from the absorbance diagram that both Pdots had an obvious absorption peak at 1,064 nm and had good performance in PTT of NIR-II (Figure 7E). In addition, S-Pdots showed low cytotoxicity and high cell viability in the *in vitro* cytotoxicity investigation. It has been verified that S-Pdots can rapidly ablate cancer cells and make it faster as the intensity of radiation increases. The 4T1 cell line was used to investigate the effect of S-Pdots *in vivo*. Mice were divided into four groups 1) phosphate buffer solution (PBS); 2) PBS + laser (1,064 nm, 0.5 W cm^−2^, 5 min); 3) S-Pdots; 4) S-Pdots + laser (1,064 nm, 0.5 W cm^−2^, 5 min). The temperature of the S-Pdots + laser group rose rapidly to 55^o^C within 5 minutes. Compared with the other three groups, the rapid temperature rise provided good conditions for PTT. Photos of the mouse tumor showed that tumor growths in this group were successfully suppressed (Figure 7F), while there was no significant change in the volume of the other three groups. In addition, the biological distribution and histological conditions of S-Pdots were further explored, and it was found that the experimental dose of S-Pdots did not affect the body of the mice, and that the concentration in the blood was minimal. It can be seen that S-Pdots have good biocompatibility, excellent photostability, and improvement in particle size leads to efficient tumor cell ablation and rapid excretion from mice. The overall performance enabled it to be a candidate of photothermal agents in clinical treatment. In addition, SPN (Jiang et al., 2018a; Zheng et al., 2020) and conjugated polymers (Song et al., 2020) are also widely used in the treatment of breast cancer.

In breast cancer treatment, two-dimensional materials can also be used as photothermal agents for PTT. Han et al. (2018) used a multifunctional sol-gel chemical method to construct a unique “therapeutic mesoporous” layer on the surface of two-dimensional Nb_2_C nanosheets, which showed a good photothermal effect when treating 4T1 breast cancer in mice. It also provides an effective strategy for the surface engineering of two-dimensional MXenes. In addition, there are numerous 2D materials used for photothermal treatment of breast cancer, such as Nb_2_C NSs (Lin et al., 2017a) and Ta_4_C_3_ SP (Lin et al., 2018) prepared by Lin et al., and Ti_2_N-MXene QDs (Shao et al., 2020a) prepared by Shao et al. Different from ordinary 2D materials, Lin et al. prepared a PVP particle system loaded with 2D Nb_2_C nanosheets (MXene) (Lin et al., 2020b). The surface of this material is micro-needle-like, as shown in Figure 7G. It has the characteristics of low invasiveness and high drug delivery efficiency on the surface. In addition, the material showed a strong absorption peak between 1,000–1,350 nm (Figure 7H), indicating its potential as a photothermal agent. In the experiment, the material and 4T1 cells were cultured *in vitro* to verify the high photothermal conversion performance and photothermal ablation ability *in vitro*. Next, *in vivo* efficacy of MXene-based MN patches in superficial tumors was observed. The temperature chart (Figure 7I) clearly showed the astonishing speed of temperature rise. In less than 2 min, the MN + laser group quickly raised the body temperature of mice to about 70°C, with excellent photothermal ablation and photothermal conversion efficiency. Among the four groups of mice, the tumor volume of the mice in the MN + laser group was smaller than that of the other groups, and after treatment the tumors in the mice almost disappeared, indicating its preliminary feasibility as a photothermal agent. In short, the minimally invasive and safe features of the microparticle system are expected to make it the preferred choice of photothermal agents for the treatment of superficial cancers.

In spite of the increasingly extensive applications of gold nanomaterials as photothermal agents, it should be noted that gold nanomaterials with large size will cause strong light scattering and reduce the light absorption effect. Zhou et al. prepared Au plasmonic blackbody (AuPBs) with controllable size through a one-pot synthesis method (Figure 7J; Zhou et al., 2018). The absorption spectrum of AuPBs is between 400–1,350 nm (Figure 7K), Unlike other NIR-II materials that use its deep penetration characteristics, the therapeutic effect of AuPBs under NIR-II is mainly based on NIR-II’s higher maximum allowable exposure (MPE) characteristics, which is better than that of NIR-I. Mice with 4T1 tumors were used in the *in vivo* treatment experiment. The AuPB + laser group showed a rapid temperature rise after treatment, which illustrated the advantages and feasibility for PTT (Figure 7L). It was shown in the tumor volume change curve in mice (Figure 7M), the AuPB + laser group effectively resisted tumor growth, and the tumor almost disappeared later in the treatment, exhibiting satisfactory treatment effect. The blackbody property enabled AuPB with good photothermal properties and was a promising photothermal agent. In addition, Jin et al. (2021) prepared corn-like Au/Ag NRs, also showed good therapeutic effect for breast cancer treatment in mice.

**FIGURE 8:**
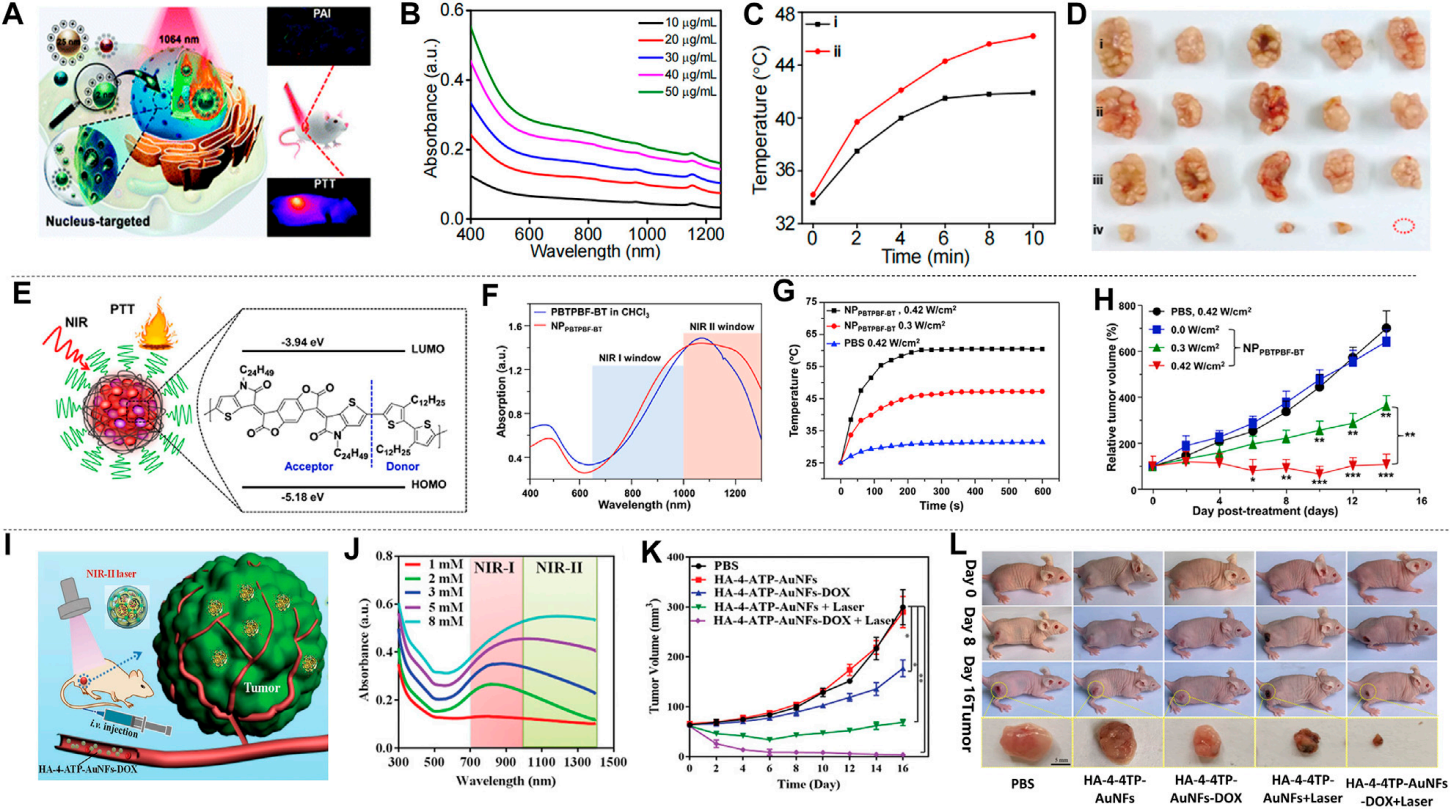
Treatment effect of mice with MCF-7 and MDA-MB-231 breast cancer cells. **(A)** Graph of nucleus-targeting ultrasmall CS-RuO_2_ NPs and for PTT under NIR-Ⅱ laser. **(B)** UV-vis-NIR absorption spectra of ultrasmall CS-RuO_2_ NPs at varying concentrations. **(C)** Temperature of tumor in live mice in two groups: 1) PBS + NIR-Ⅱ, 2) ultrasmall CS-RuO_2_ NPs + laser. **(D)** Representative digital photos of tumor under four groups treatment: 1) PBS, 2) PBS + laser, 3) ultrasmall CS-RuO_2_ NPs, 4) ultrasmall CS-RuO_2_ NPs + laser (reproduced from (Liu et al., 2020) with permission from Royal Society of Chemistry). **(E)** Schematic illustration of the preparation of NP_PBTPBF-BT_ for PTT. **(F)** Absorption spectra of nanoparticle NP_PBTPBF-BT_ and PBTPBF-BT in CHCl_3_. **(G)** Temperature change line during the three groups treatment. **(H)** The MDA-MB-231 tumor growth curves of different groups of mice (reproduced from (Cao et al., 2018) with permission from Elsevier BV). **(I)** HA-4-ATP-AuNFs-DOX for PTT. **(J)** UV–vis–NIR spectra of AuNFs with various HAuCl_4_ concentration. **(K)** The tumor volume changes of five groups’ treatment. **(L)** Photos of mice in five groups during three treatment period and tumor photo after treatment (reproduced from (Wang et al., 2020) with permission from Wiley-VCH Verlag).

In addition to the 4T1 breast cancer cell line, the MCF-7 cell line and MDA-MB-231 cell line are also frequently used in the study of breast cancer treatment. Cheng et al. (2019) prepared two-dimensional TeO_2_/(NH_4_)xWO_3_ for MCF-7 breast cancer treatment, and Zhang et al. (2020d) prepared TAT-Pd@Au/Ce6/H-MnO_2_. Liu et al. (2020b) made size modification and chemical modification of RuO_2_ NPs, and prepared nuclear-targeted ultrasmall CS-RuO_2_ NPs using one-pot synthesis method (Figure 8A). CS-RuO_2_ NPs had appropriate size and biological stability. The nuclear targeting effect of CS effectively helped CS-RuO_2_ NPs quickly enter the cancer cell nucleus, and showed excellent photothermal properties under NIR-II irradiation. Under 1,064 nm irradiation, CS-RuO_2_ NPs of different concentrations showed strong absorbance in NIR-II (Figure 8B). Mice carrying MCF-7 breast cancer cells were used to study the performance of CS-RuO_2_ NPs in photothermal treatment at low temperature. It can be seen from the temperature change graph that the difference in temperature rise between 1) PBS group and 2) CS-RuO_2_ NPs + laser group was not significant (Figure 8C). From the change of tumor volume (Figure 8D), it was found that the CS-RuO_2_ NPs + laser group has a very obvious anti-cancer advantage, in which group the growth of the tumor was significantly inhibited. In general, the material prepared by Liu et al. has shown its outstanding advantages in low-temperature PTT.

Improving the absorbance of nanomaterials is an important research direction of nanomaterials. Cao et al. prepared a thieno-isoindigo derivative-based D-A polymer (PBTPBF-BT) with high light absorption (Figure 8E; Cao Z. et al., 2018). Figure 8F showed its strong absorbance in NIR-II that indirectly improves optical heat conversion efficiency. In the treatment of breast cancer, four groups (*n* = 5) of nude mice carrying MDA-MB-231 tumors were observed: 1) PBS + NIR-II irradiation (0.42 W cm^−2^, 10 min). 2) NP_PBTPBF-BT_ + NIR-II irradiation (1,064 nm, 0.0 W cm^−2^, 10 min). 3) NP_PBTPBF-BT_ + NIR-II irradiation (1,064 nm, 0.3 W cm^−2^, 10 min). 4) NP_PBTPBF-BT_ + NIR-II irradiation (1,064 nm, 0.42 W cm^−2^, 10 min). The temperature curve showed the fastest temperature rise in the group of NP_PBTPBF-BT_ at 0.42 W cm^−2^ intensity of 1,064 nm irradiation (Figure 8G). After 14 days of treatment, the fourth group shows the best treatment effect, and the tumor volume gradually decreased (Figure 8H), while the other groups cannot effectively inhibit tumor growth. The photothermal conversion efficiency of this material is 66.4% under 1,064 nm irradiation, which is outstanding among similar materials.

Wang et al. (2020c) prepared HA-4-ATP-AuNFs-DOX through a simple and green approach. HA-4-ATP-AuNFs-DOX has high biocompatibility, strong light absorption and high photothermal conversion, and the mesoporous cavity of AuNFs can load a large number of anti-cancer drugs for chemotherapy, which is advantageous for cancer treatment (Figure 8I). From the absorption graph of AuNFs prepared with different concentrations of HAuCl_4_ (Figure 8J), it can be seen that AuNF materials had good absorption performance in NIR-II. Among them, AuNFs prepared from HAuCl_4_ with a concentration of 8 × 10^–3^ mM had the highest absorption peak in NIR-II. The green preparation method made it non-cytotoxic. *In vivo* experiments illustrated the therapeutic effects of five groups of different conditions on mice bearing MDA-MB-231 tumors. As can be seen from the tumor volume change curve (Figure 8K), HA-4-ATP-AuNFs-DOX + laser group not only effectively inhibited tumor growth, but also completely eliminated the tumor at the late stage of the treatment. Figure 8L showed the pictures of the mice during the treatment and the tumor block diagram after the treatment, which illustrated the treatment effectiveness of HA-4-ATP-AuNFs-DOX + laser group. The ability of HA-4-ATP-AuNFs-DOX to eradicate tumors makes it promising in clinical treatment.

The PTT guided by NIR-II has shown its advantages in the treatment of breast cancer. In the research of PTT of breast cancer, more suitable photothermal agents can be found by modifying the physical or chemical properties of the materials. Various materials with different performances in the process of treating breast cancer have been synthesized in terms of biocompatibility. Photothermal agents with excellent performance in various aspects such as cytotoxicity, photothermal conversion performance, etc. are the direction of efforts.

### Cervical Cancer

Cervical cancer is the deadliest cancer for women in the world. Globally, there are about 500000 new cases of cervical cancer each year, accounting for 5% of all new cancer cases. According to the World Health Organization, if no further action is taken, the number of new cervical cancer cases will increase from 570000 to 700000 annually from 2018 to 2030, and the number of deaths each year is expected to increase from 311000 to 400000. Scientists have made many attempts and efforts to develop more efficient photothermal reagents for the treatment of cervical cancer. Gao et al. (2019) reported heterostructures of coated Au nanosheet (Au NPL@TiO_2_) coated with titanium dioxide. This material has a high photothermal conversion efficiency of 42.05% under 1,064 nm laser irradiation. Animal experiments also showed a significant tumor elimination effect. Synergistic PTT/sonodynamic therapy showed the best anti-tumor effect. This work provides a reference for the use of 2D gold nanosheet core/TiO_2_ shell nanostructures for multifunctional cancer treatment.

**FIGURE 9:**
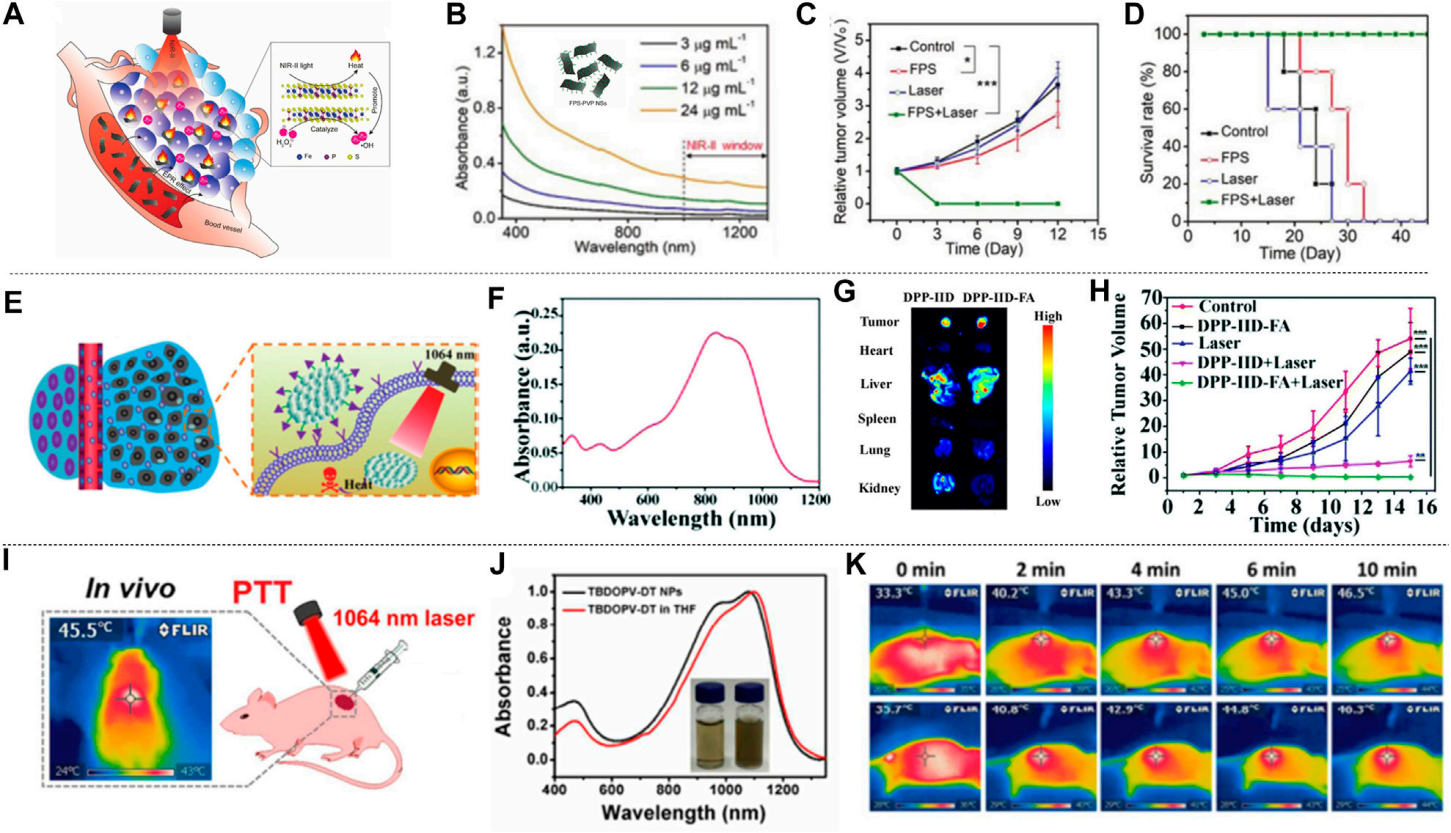
Photothermal treatment of cervical cancer. **(A)** FPS-PVP NSs are used in the synergistic treatment of cancer chemodynamic therapy and PTT in the NIR-Ⅱ. The illustration is the bat model of FPS. **(B)** UV-vis-NIR absorbance spectra of FPS-PVP NSs at different Fe concentrations (3, 6, 12, and 24 μg ml^−1^). **(C)** Growth curves of mouse tumors at 12 days after different treatments (*n* = 5). **(D)** The survival rate of mice after 12 days of different treatments (reproduced from (Zhang et al., 2020) with permission from Wiley-VCH Verlag). **(E)** Schematic diagram of DPP-ⅡD-FA nanoparticles for photothermal treatment of superficial tumors. **(F)** Absorption spectrum of DPP-ⅡD-FA nanoparticles dispersed in PBS. **(G)** *Ex vivo* fluorescence image of mouse main organs 24 h after DPP-ⅡD-FA nanoparticle injection. **(H)** Relative tumor volumes after various treatments (reproduced from (Wei et al., 2018) with permission from Royal Society of Chemistry). **(I)** Application of TBDOPV-DT NPs in PTT *in vivo*. **(J)** Absorption spectra and picture (inset) of TBDOPV-DT NPs (water, right) and TBDOPV-DT (THF, left). **(K)** The whole-body IR images of mice after injection of TBDOPV-DT NPs intratumorally (0.56 mg kg^−1^) and irradiated by 1,064 nm laser (0.90 W cm^−2^) or intravenously (1.94 mg kg^−1^) and irradiated by laser (1.30 W cm^−2^) (reproduced from (Sun et al., 2018) with permission from American Chemical Society).

Zhang et al. (2020b) performed liquid exfoliation of FePS_3_ (FPS) nanosheets (NSs) and surface modification with poly (vinylpyrrolidone) (PVP) molecules (FPS-PVP NSs) to improve the dispersion and stability of the nanosheets, thereby preparing a new type of 2D nano platform (Figure 9A). FPS-PVP NSs showed good absorption capacity in NIR-II, which can meet the conditions required for photothermal treatment (Figure 9B), and has a photothermal conversion efficiency of up to 43.3% in NIR-II. It exhibited significant Fenton catalytic activity, which can be further enhanced synergistically through its excellent photothermal effect. HeLa tumor-bearing mice were used to explore the effect of FPS-PVP NSs in cancer treatment *in vivo*. The mice were randomly divided into four groups: 1) control; 2) laser; 3) FPS-PVP NSs; 4) FPS-PVP NSs + laser group. Without laser irradiation, the tumor size of the mice in the control group and FPS-PVP NSs group increased significantly. After irradiated with near-infrared laser (1 W cm^−2^, 1,064 nm, 10 min) for 24 h, the temperature of the tumor in the FPS-PVP NSs + laser group rose rapidly to ≈53˚C, indicating the effective accumulation and excellent photothermal conversion effects in tumors. As shown in Figure 9C, within 12 days after treatment, FPS-PVP NSs showed a certain inhibitory effect on tumor growth, and the tumor was completely eradicated when irradiated by an external NIR laser. The high temperature of about 53˚C induced by PTT can cause fatal damage to cancer cells and play a major role in the eradication of tumors. Chemodynamic therapy (CDT) induced by its Fenton properties plays a supporting role. After treatment, the survival rate of mice was significantly improved without recurrence (Figure 9D). The 2D nanoplatform is composed of biocompatible elements (iron, phosphorus, sulfur) that can be removed from the body. Its excellent biological safety further ensures its great potential in tumor photothermal treatment.

Polymer materials have natural advantages of biocompatibility due to their completely organic nature. A variety of polymer materials have been developed as photothermal agents for cervical cancer. Wei et al. (2018) designed and synthesized a new diketopyrrolopyrrole polymer (P(AcIIDDPP)), and then prepared polymer nanoparticles (DPP-IID-FA) by the emulsion method (Figure 9E). The polymer has strong light absorption in NIR-I and NIR-II (Figure 9F). *Ex vivo* imaging analysis of tumors and other major organs of mice proved the ability of DPP-IID-FA nanoparticles to accumulate in tumors. As shown in Figure 9G, the labeled nanoparticles inevitably entered the metabolic organs such as the liver, and a considerable amount of DPP-IID-FA nanoparticles still significantly accumulated in the tumor 24 h after injection. After 10 min (1,064 nm, 1 W cm^−2^) irradiation, the local temperature of the tumors of mice injected with DPPIID-FA nanoparticles quickly rose to 57.2˚C, while the DPP-IID treatment group only increased to 45.3 ± 1°C. In contrast, irradiating with laser without nanoparticles caused only a slight temperature increase. These results indicated the excellent heat-generating and targeting ability of the prepared nanoparticles. The tumor size was measured every 2 days during the treatment to evaluate the treatment efficiency (Figure 9H). The tumors of the DPP-IID-FA injection +1,064 nm laser irradiation group were completely suppressed, and there was no relapse during the 14 days observation period. At the same time, the tumor growth of mice in the DPP-IID injection +1,064 nm laser irradiation group showed a certain inhibitory effect, while the tumor volume was rapidly increased in the group injected with saline and DPP-IID-FA without 1,064 nm laser irradiation. This indicates that the nanoparticle has a strong cancer cell killing ability and excellent PTT effect, and is expected to become a new type of ultra-wide-spectrum anti-cancer drug for NIR-II.

Sun et al. (2018), using 2,2-dithiophene as the donor and thiophene-fused benzo-difuran dione as the acceptor, developed a new type of NIR-II photothermal nano-agent. TBDOPV-DT nanoparticles showed very strong absorption in the NIR (Figure 9J). The tumor suppression effect was evaluated with HeLa tumor-bearing nude mice subjected to *in vivo* phototherapy under 1,064 nm laser irradiation (Figure 9I). The infrared thermal image showed that laser irradiation + intratumoral/intravenous (it/iv) injection of TBDOPV-DT nanoparticles can achieve a rapid increase in temperature (Figure 9K). After 20 days treatment, the mice were sacrificed and the tumors were resected. The results showed that there were no tumors in the it/iv + laser group, indicating successful photothermal ablation of the tumors with the nanoparticles. Changes in body weight can be used to assess the potential adverse effects of photothermal agents. Only the mice in the iv injection + laser group showed an insignificant decrease on body weight in the first 2 days but recovered rapidly afterward. In addition, the weight of the mice in the treatment group was slightly higher than that of the control group, indicating that these treatments had no obvious toxic effects on the animals. The high photothermal conversion efficiency (50%), excellent anti-cancer effect, and good biological safety of TBDOPV-DT nanoparticles provide a reference for the design and development of organic polymer photothermal agents.

Lei et al. (2019) prepared Ni_9_S_8_ NPs with full-spectrum absorption (400–1,100 nm) in the NIR. Its photothermal conversion efficiency at 1,064 nm can be as high as 46.0%, which is enough to destroy cancer cells and ablate solid tumors. *In vivo* PTT had also achieved effective inhibition of tumors, so it can be used as an effective and safe photothermal agent in the NIR-II. Li et al. coated Ag_2_S nanoparticles (NPs) with mesoporous silica (Li et al., 2021), then loaded a low-oxygen active prodrug (tirapazamine) inside the mesoporous silica, and coated the surface with glucose oxidase (GOx) and the synthesized Ag_2_S@MSN-TGF nano-platform synergizes with starvation, hypoxic active prodrug therapy, and PTT effect, and shows excellent tumor inhibition *in vivo*. Compared with a single treatment model, it significantly improves the anti-tumor effectiveness.

### Liver Cancer

Liver tumors have a low surgical resection rate, high recurrence rate, and strong invasiveness. There is an urgent need for effective tumor ablation with low side effects. PTT has great advantages in this regard. In the early years, Shao et al. (2020b) used triphenylamine (TPA) as the donor and benzo [1, 2-c:4, 5-c’] bis ([1, 2, five] thiadiazole (BBT) as the acceptor to synthesize three D-A-D conjugated small molecule nanoparticles CSM0-2. The number of thiophene bridges was 0, 1, 2, respectively. Among them, CSMN2 has the superior photothermal ability of deep tissue in the NIR-II. HuH-2 liver cancer cells were significantly destroyed after 1,064 nm laser irradiation in its presence. Small-molecule carbon nanotubes have a clear chemical structure and are easy to prepare and purify. Therefore, they have considerable prospects for clinical translation as nanomedicine.

**FIGURE 10:**
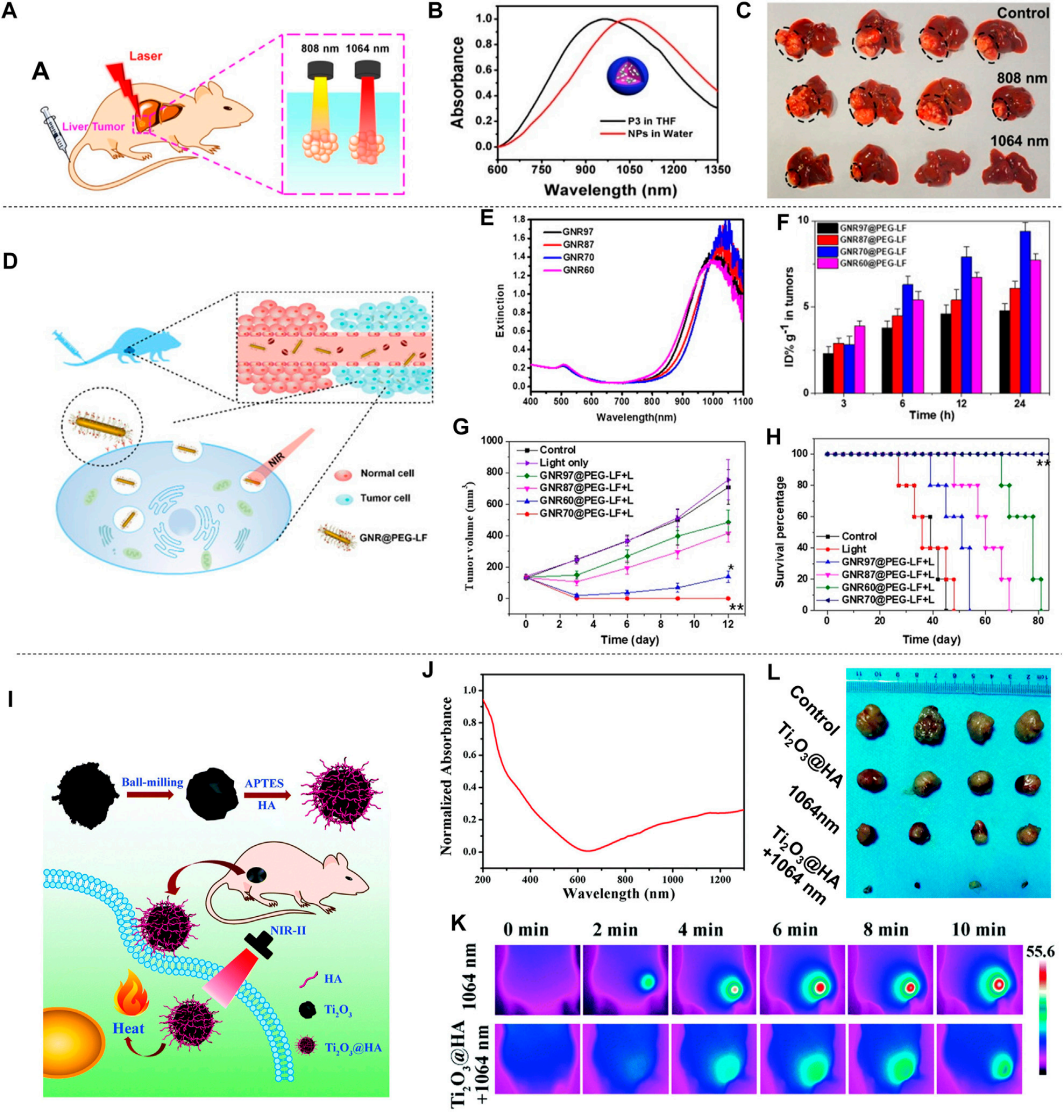
Photothermal treatment of liver cancer. **(A)** Schematic diagram of PTT of liver cancer *in situ* with SPNs under 808 or 1,064 nm laser irradiation. **(B)** Absorption spectra of four semiconducting polymers (P1-P4) in THF. **(C)** Photograph of liver of tumor-bearing mice resected 17 days after treatment. Black circles indicate tumors in the liver (reproduced from (Sun et al., 2019) with permission from American Chemical Society). **(D)** Schematic diagram of the distribution of GNR@PEG-LF *in vivo* and its use in photothermal treatment of cancer. **(E)** UV-vis-NIR spectra of GNRs@CTAB with different widths and lengths. **(F)** Histogram of the concentration of GNR@PEG-LF in tumors. **(G)** Tumor growth inhibition curves in mice injected with different GNRs and irradiated with 980 nm light (0.5 W cm^−2^) for 3 min. **(H)** The Kaplan-Meier graph shows the percentage of animals surviving the study over time (reproduced from (Yang et al., 2020) with permission from Academic Press Inc.). **(I)** Schematic diagram of the preparation of Ti_2_O_3_@HA and its PTT for superficial tumors. **(J)** Absorption spectrum of Ti_2_O_3_@HA nanoparticles in PBS. **(K)** Infrared thermal image of HepG-2 tumor-bearing mice injected with PBS or Ti_2_O_3_@HA nanoparticles and irradiated with a 1064 nm laser (5 min at a power density of 1.0 W cm^−2^). **(L)** Tumor images of each group after 14 days of different treatments (reproduced from (Zeng et al., 2018) with permission from Royal Society of Chemistry).

The excellent absorption ability of semiconducting polymers in the NIR-II aroused widespread interest. Sun et al. (2019a) prepared SPNs by a ternary copolymerization method. The structure is shown in the inset in Figure 10B. The nanoparticles have broad absorption in both the NIR-I and the NIR-II (Figure 10B). Nanoparticles prepared from semiconducting polymers were used for the treatment of liver cancer *in situ* after laser irradiation (Figure 10A). The mice were divided into three groups: 1) control; 2) 808 nm laser + SPNs; 3) 1,064 nm laser + SPNs. The results showed that the mice in the 1,064 nm laser + SPNs group had a higher temperature than the group that used 808 nm laser, which indicated that the SPNs had a higher photothermal conversion efficiency under 1,064 nm laser. After 17 days of treatment, the tumor-bearing liver was resected (Figure 10C). Large tumors appeared in the control group and the 808 nm laser + SPNs group. Two tumors in the livers of the 1,064 nm group were much smaller than the formers, and the tumors in the other two livers completely disappeared. The polymer nanoparticles effectively inhibited or completely eradicate the liver tumors. Wei et al. prepared a new type of narrow bandgap conjugated polymer-based near-infrared diagnostic nanoparticles with a photothermal conversion efficiency as high as 65% (Wei et al., 2020). The obtained NPs_PBBTDTS_-Aptamer nanoparticles (BDA NPs) showed good ability to target to and accumulate in human liver cancer cells *in vivo*. BDA NPs completely ablated the liver tumors with excellent light-to-heat conversion efficiency and good biological safety.

Gold nanomaterials have adjustable near-infrared LSPR peaks and are a popular choice for photothermal therapy. Yang et al. (2020) prepared a series of GNR with similar aspect ratios and LSPR peaks, and modified with polyethylene glycol and tumor-targeting ligand lactoferrin. Then HepG2 liver cancer cells were used for verifying their anti-cancer effect of PTT (Figure 10D). The GNRs have a strong absorption capacity in the NIR-II (Figure 10E). Among them, GNR with a medium size (length 70 nm, width 11.5 nm) and PET-modified surface (GNR70@PEG modification) showed the fastest cell internalization and the best *in vitro* photothermal results on liver cancer cells. Due to the synergy of surface coating and size, GNR70@PEGLF also showed a long circulation time and the highest tumor accumulation in the body (Figure 10F). The tumor ablation ability under 980 nm light irradiation was verified on a mouse xenograft model (Figure 10G), and the results showed good anti-cancer PTT effects in the NIR-II region. Significantly, the Kaplan-Meier survival curve showed that during the experiment (Figure 10H), the group of GNR70@PEG-low frequency + light had a 100% survival rate, while the median survival of the control group, the light-only group, GNR97@PEG-low frequency + light group, GNR87@PEG-low frequency + light group and GNR60@PEG-low frequency + light group was determined to be 36, 39, 45, 57 and 69 days, respectively. This result indicated that the complete eradication of tumors can really benefit the long-term survival of mice, although the strong photothermal effect is helpful for tumor treatment. GNR70@PEG-LF completely destroyed the tumor without recurrence after one treatment (iv injection + light irradiation). It has a better therapeutic effect than other GNRs, which is attributed to the synergy of proper size and ligand modification leading to high tumor accumulation. This research provided new information for the design of tumor-targeting nanomaterials for PTT. Zhang et al. (2019b) proposed a gold nanocarrier-DTPP nanocarrier that conjugated the chimeric peptide DTPP to the surface of a gold nanocarrier through a gold-sulfur bond and proved the nanocarrier had great inhibitory effect on H22 liver cancer cells. Combined with real-time apoptosis imaging and auxiliary photodynamic therapy, the anti-tumor effect is further enhanced.

Zeng et al. (2018a) prepared Ti_2_O_3_ nanoparticles by ball milling and modified them with HA to improve biocompatibility and targeting. Then Ti_2_O_3_@HA-FITC was intravenously injected into HepG-2 tumor-bearing mice. The mice were treated with photothermal treatment (Figure 10I). The prepared nanoparticles have strong light absorption in NIR-II (Figure 10J). The mice were divided into four groups (*n* = 4): 1) control group; 2) 1,064 nm laser irradiation alone; 3) Ti_2_O_3_@HA nanoparticles alone; 4) Ti_2_O_3_@HA + 1,064 nm laser irradiation. Twelve hours post injection, the mice in groups 2) and 4) were exposed to 1,064 nm laser irradiation. As shown in Figure 10K, mice injected with Ti_2_O_3_@HA nanoparticles showed a significant temperature increase. Under the NIR-II laser irradiation (1,064 nm, 1.0 W cm^−2^), it reached 55.6 ± 1°C after 5 min, which is enough to kill cancer cells. In contrast, with only laser irradiation, the local temperature of the tumor only increased to about 42.7°C. These results proved the superior heat production capacity and highly targeted accumulation potential of Ti_2_O_3_@HA nanoparticles, which provided the possibility for photothermal ablation of tumors *in vivo*. After 14 days treatment, tumors in mice were resected (Figure 10L). Only the Ti2O3@HA + 1,064 nm laser irradiation group showed effective tumor growth inhibition, which almost completely ablated the tumor, while tumors in the other groups grew rapidly showing no treatment effect. These results clearly proved the excellent PTT therapeutic effect of Ti_2_O_3_@HA *in vivo*. The excellent biological safety of Ti_2_O_3_@HA in the body proved that it can be used as an excellent new PTT agent for the treatment of cancer in the NIR-II region safely. Du et al. (2017) constructed a multifunctional nano-therapeutic agent that uses amphiphilic D-α-tocopherol polyethylene glycol 1,000 succinate (TPGS-Cu_3_BiS_3_) to functionalize bimetallic chalcogenide nanocrystals (NCs). TPGS-Cu_3_BiS_3_ is both a radiosensitizer and a photothermal agent, and obtained excellent therapeutic effects in the combination of radiation therapy and PTT. TPGS-Cu_3_BiS_3_ NCs provided a new strategy for the treatment of deep tumor cells.

Due to the complexity of liver cancer treatment, other photothermal reagents and more effective treatment strategies still need to be explored.

### Oral Cancer, Sarcoma, and Colon Cancer

**FIGURE 11:**
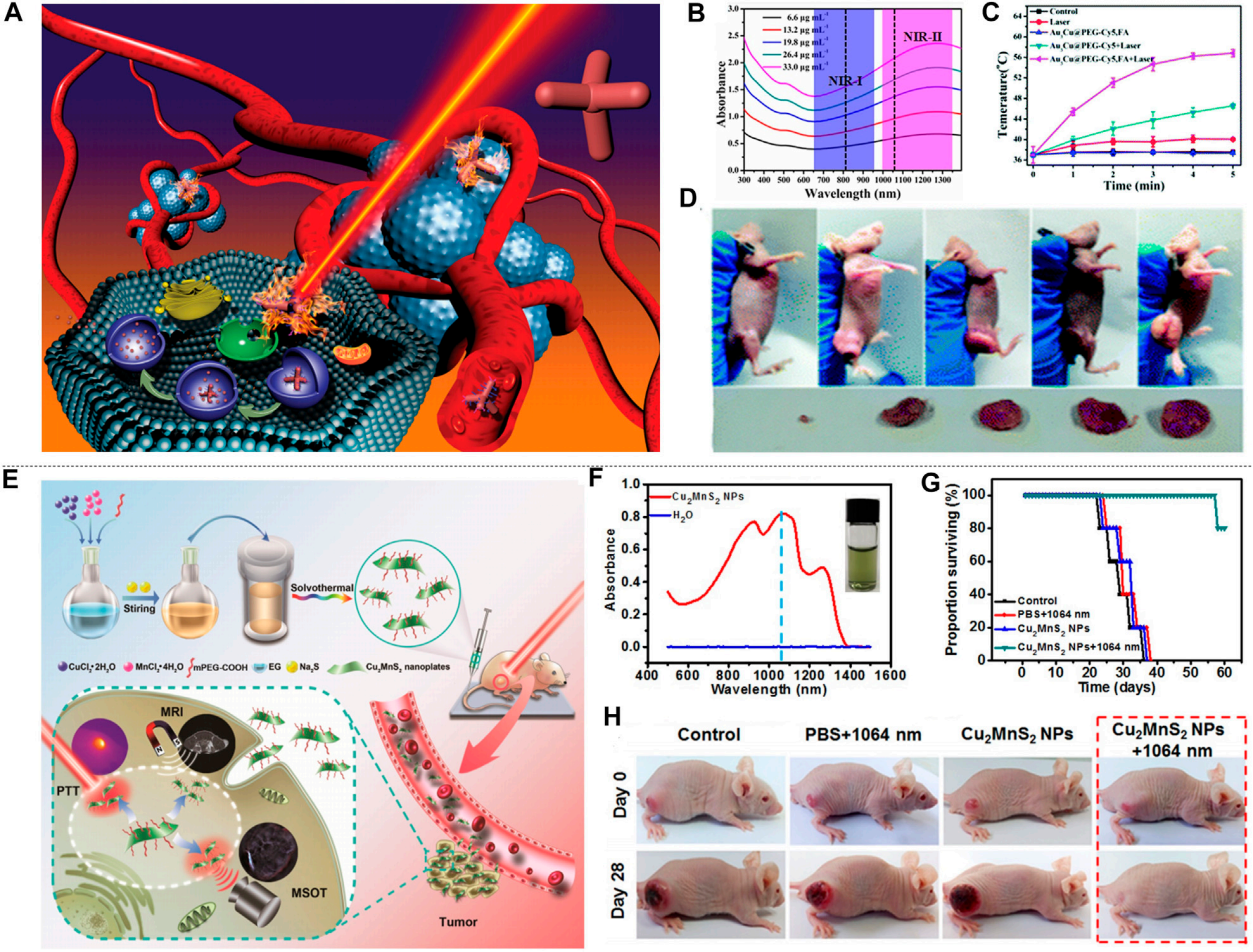
PTT of oral cancer, sarcoma cancer and colon cancer. **(A)** System diagram of Au_3_Cu TPNCs. **(B)** Absorption spectrum of Au_3_Cu TPNCs. **(C)** The temperature change of tumor in mice with KB. **(D)** the comparison of tumors in five groups of mice (reproduced from (Wang et al., 2018) with permission from Royal Society of Chemistry). **(E)** The synthesis of Cu_2_MnS_2_ NPs and the process of photothermal treatment of mice with S180. **(F)** Absorption spectrum of Cu_2_MnS_2_ NPs. **(G)** Survival rate of four groups of mice. **(H)** Comparison of tumor size of four groups of mice (reproduced from (Ke et al., 2017) with permission from Ivyspring International Publisher).

In the study of cancer cells, researchers have discovered a lot of materials for photothermal treatment of oral cancer, sarcoma and colon cancer in NIR-I (Shan et al., 2011; Wang et al., 2011; Su et al., 2012; Xiao et al., 2012; Wang et al., 2013; Liu et al., 2018; Wang et al., 2020e), and have obtained good results utilizing the photothermal properties of the materials. In order to improve the effect of PTT, researchers also studied some nanomaterials in NIR-II. Although oral cancer is uncommon and its cause is unknown, some common factors in daily life can increase the risk of oral cancer, such as smoking, drinking, eating less fruits and vegetables, etc., and it is necessary to study the treatment of oral cancer. For the treatment of oral cancer, Wang et al. (2018c) used seed-mediated growth method to prepare Au_3_Cu TPNCs (Figure 11A). It has a broad absorption peak at 700–1,400 nm, and showed good photothermal stability (Figure 11B). The Au_3_Cu TPNCs were modified with the Cy5, PEG and FA to prepare Au_3_Cu@PEG-Cy5, FA, which improved the biological stability of Au_3_Cu TPNCs and endowed it with targeting effect against tumor cells over-expressing folate receptor (FR). Under the same laser irradiation, Au_3_Cu@PEG-Cy5, FA killed a higher number of KB cells than Au_3_Cu@PEG-Cy5, which proved that Au_3_Cu@PEG-Cy5, FA can achieve the accumulation of more Au_3_Cu TPNCs into KB cells. The Au_3_Cu@PEG was dissolved in a PBS similar to the tumor microenvironment at pH = 5, and the absorption spectrum of this solution was observed for 8 days. Compared with the absorption peak of 1,278 nm on the first day, the absorption peak on the eighth day had been blue shifted to 1,180 nm, indicating the degradability of Au_3_Cu@PEG-Cy5, FA. In order to verify the PTT effect of Au_3_Cu@PEG-Cy5, FA, 25 mice with KB were divided into five groups accepting intravenous injection: 1) 1,064 nm laser + Au_3_Cu@PEG-Cy5, FA; 2) 1,064 nm laser + Au_3_Cu@PEG-Cy5; 3) Au_3_Cu@PEG-Cy5, FA; 4) saline; 5) 1,064 nm laser + saline. After 5 min of laser irradiation, the local temperature of the tumors in the first group rose from 37°C to 56.81°C, which exceeded the 42°C required to kill cancer cells and was much higher than the other four groups (Figure 11C). It shows that Au_3_Cu@PEG-Cy5, FA has good photothermal properties. The body weight of the first group didn’t decrease, proving the low toxicity of Au_3_Cu@PEG-Cy5, FA. The tumors in the five groups of mice were taken out, and it was found that the tumor volume in the first group was the smallest, proving that Au_3_Cu@PEG-Cy5, FA has a better PTT effect (Figure 11D). Finally, the main organs of the first group of mice (heart, liver, spleen, stomach, kidney, viscera, lung, etc.) were taken out, and no obvious damage was found, indicating that Au_3_Cu@PEG-Cy5, FA caused little damage to the main organs and had good biocompatibility. Therefore, the Au_3_Cu@PEG-Cy5, FA could be successfully used for PTT. Zhang et al. (2020a) modified the aggregated hybrid gadolinium (Gd) and CuS nanogel platform (NG) by an inverse emulsion method through a Michael-addition crosslinking reaction using polyethylenimine (PEI) and folic acid (FA) to prepare Gd/CuS@PEI-FA-PS NGs. The absorption spectrum of Gd/CuS@PEI-FA-PS NGs is 600–1,100 nm and has good photothermal properties. Mice bearing KB tumors with high folate receptor expression (KB-HFAR)/low folate receptor expression (KB-LFAR) were used to verify the targeting effect of Gd/CuS@PEI-FA-PS NGs. Gd/CuS@PEI-FA-PS NGs dispersed in PBS solution were injected subcutaneously into the right thigh of mice. Gd was detected in both tumors, and the higher content of Gd in the KB-HFAR group proved high targeting effect of Gd/CuS@PEI-FA-PS NGs mediated by FA. In order to further explore the PTT effect of Gd/CuS@PEI-FA-PS NGs, mice with KB cells were tested by PTT. It was found that the tumors in KB-HFAR group injected with Gd/CuS@PEI-FA-PS NGs and irradiated with 1,064 nm laser were completely eradicated. Together with no damage to the main organs of these mice, it is proved that Gd/CuS@PEI-FA-PS NGs has biocompatibility and good PTT effect. Sarcoma is mainly derived from connective tissue, characterized by rapid development, short course of disease and rapid deterioration. It has a relatively high incidence among young people. Therefore, how to inhibit the growth of sarcoma is a necessary research direction. Ke et al. (2017) synthesized Cu_2_MnS_2_ NPs with a Cu:Mn:S ratio of 2.03:1:1.99 by a one-pot solvothermal method, and used a dual-mode magnetic resonance imaging (MRI)/multispectral photoacoustic tomography (MSOT) state imaging guided PTT to treat sarcoma (Figure 11E). The absorption peaks of Cu_2_MnS_2_ NPs in NIR-II located at 1,075 nm and 1,260 nm, and the photothermal conversion efficiency was shown in Figure 11F. In order to study the PTT of Cu_2_MnS_2_ NPs *in vivo*, mice with S180 were divided into four groups accepting intravenous injection: 1) No treatment; 2) 1,064 nm laser + PBS; 3) Cu_2_MnS_2_ NPs; 4) 1,064 nm laser + Cu_2_MnS_2_ NPs. Within 3 min, the temperature of the tumors in the fourth group rose from 33 to 55°C, indicating that Cu_2_MnS_2_ NPs have good photothermal conversion properties. After 37 days of follow-up observation, only the fourth group of mice did not lose weight and survived (Figure 11G). During 28 days of observation, it was found that only the tumors of the fourth group of mice were completely eradicated, proving that Cu_2_MnS_2_ NPs have a good photothermal treatment effect (Figure 11H). The presence of Mn in the main organs of the surviving mice was also assayed. After 7 days, Mn had been completely eliminated from these main organs, which proved that Cu_2_MnS_2_ NPs did not damage other organs of the mice and had good biocompatibility.

Colon cancer is a kind of malignant tumor located in the digestive tract. It is aggressive to many internal organs and tissues, which can cause great harm to the body of the patient. The lethality rate of colon cancer is really high, so it is necessary to use PTT to suppress this tumor. Ding et al. (2014) synthesized dual-plasma Au-Cu_9_S_5_ NPs by seed-mediated growth method. It has an absorption peak at 1,100 nm. The colocalization of Au and Cu enhanced the light absorption area of Au-Cu_9_S_5_ NPs, and promised a better PTT potential in NIR-II. In order to verify the PTT effect of Au-Cu_9_S_5_ NPs on colon cancer cells, mice were divided into two groups for intravenous injection: 1) 1,064 nm laser + PBS; 2) 1,064 nm laser + Au-Cu_9_S_5_ NPs. A thermal imaging camera was used to monitor the temperature changes of the tumors in the mice. The temperature of the second group rose from 35 to 54°C within 2 min, which is much higher than that of the first group and proved that Au-Cu_9_S_5_ NPs have a strong photothermal conversion efficiency. It was found that cancer cells in the Au-Cu_9_S_5_ NPs group underwent a faster apoptosis by observing the cancer tissue stained with Hematoxylin and eosin (H and E), which indicated that Au-Cu_9_S_5_ NPs is a good PTT material. Zhang et al. (2020c) synthesized squaraine-based semiconducting polymer nanoparticles (PSQPNs-DBCO). Under 1,064 nm irradiation, the emission spectrum is at 1,150–1,500 nm, and fluorescence imaging was used to guide PTT in mice with CT26 at NIR-II. The mice with CT26 recovered, and no damage to other major organs were found, indicating that PSQPNs-DBCO has good biocompatibility and achieved good PTT effects.

### Lung Cancer and Glioma

**FIGURE 12:**
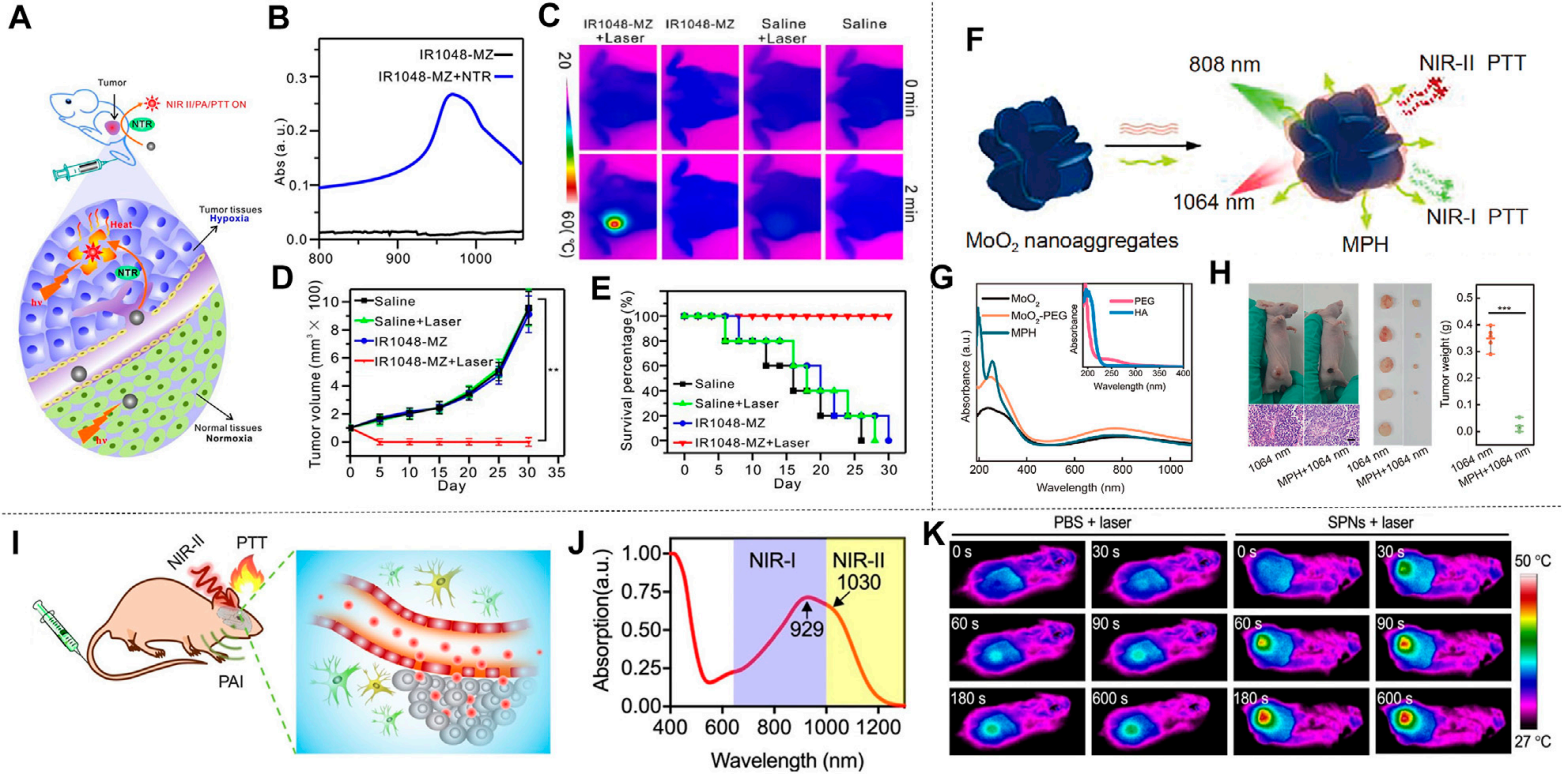
PTT of lung cancer and glioma. **(A)** IR1048-MZ as a novel NTR enzyme response single-molecule probe for NIR-Ⅱ hypoxia-activated PTT. **(B)** The absorption spectrum of IR1048-MZ when NTR is absent (black) and when NTR is present (blue). **(C)** Infrared thermal images of lung tumor-bearing mouse injected with saline or IR1048-MZ with or without 980 nm NIR laser irradiation (0.1 W cm^−2^) for 2 min. The room temperature is 23˚C. **(D)** Lung tumor growth curve after 30 days of different treatments. **(E)** Survival rate of lung tumor-bearing mice after various treatments (*n* = 5) (reproduced from (Meng et al., 2018) with permission from Ivyspring International Publisher). **(F)** Schematic diagram of NIR response to MoO_2_ nano-aggregates and their PTT applications. **(G)** UV-vis-NIR absorption spectrum of MoO_2_ nano aggregate surface-modified. **(H)** lung-tumor-bearing mouse and it’s tumors dissected after treatment, and tumor weights after 16 days of treatment (*n* = 5) (reproduced from (Guo, et al., 2020) with permission from Science China Press and Springer-Verlag GmbH Germany, part of Springer Nature). **(I)** Schematic diagram of SPNs used in NIR-Ⅱ photothermal treatment of brain tumors. **(J)** Absorption spectra of SPNs in aqueous dispersion. **(K**) The infrared thermal image of the mouse during the 10-min PTT (1,064 nm, 1 W cm^−2^). Mice treated with PBS + laser were used as controls (reproduced from (Wen, et al., 2020) with permission from American Chemical Society).

High smoking rate leads to an increase in the incidence of lung cancer, and its mortality is rising rapidly, which makes lung cancer a global burden. Therefore, the suppression of lung cancer has become an urgent need. Meng et al. (2018) synthesized the fluorescent probe IR1048-MZ as a hypoxia-triggered and nitroreductase (NTR) enzyme-responsive single-molecule probe. The IR1048-MZ was used to locate tumors and monitor tumor hypoxia as well as PTT (Figure 12A). The absorption of IR1048-MZ at 900–1,060 nm is relatively weak. After the reduction by NTR, there is a maximum absorption peak at 980 nm, which proved that IR1048-MZ is sensitive to NTR (Figure 12B). Hypoxic tumors can overexpress NTR. In order to prove that hypoxia in cancer cells caused PTT, an endogenous inhibitor (dicoumarin) was used to inhibit hypoxia for NTR. The dicoumarin was injected only into the tumor on the left leg (tumor 1), and not injected on the right leg (tumor 2), and then IR1048-MZ was injected into the two lung cancer tumors through tail vein injection. The temperature of tumor 2 rose from 30 to 58°C within 2 min, reaching the lethal temperature of the tumor cells, while the temperature of tumor 1 is still 30°C. The tumor was sectioned and it was found that the cells of tumor 2 were hypoxic, which proved that the photothermal conversion is caused by hypoxia. In order to verify the effect of PTT caused by hypoxia, mice bearing lung cancer were divided into four groups: 1) saline 2) 980 nm laser + saline 3) IR1048-MZ 4) 980 nm laser + IR1048-MZ. After irradiating with 980 nm laser for 2 min, the temperature of the fourth group increased significantly, while the other three groups hardly changed, indicating that IR1048-MZ is a good photo-thermal sensitizer (Figure 12C). After 30 days of observation, only in the fourth group, the tumor cells were completely eliminated and the mice survived, proving the feasibility of IR1048-MZ probe hypoxia-stimulated PTT (Figures 12D,E). Guo et al. prepared MoO_2_ nanomaterials by a one-pot hydrothermal method (Guo et al., 2020), and modified them with PEG and HA to obtain MoO_2_@PEG@HA (MPH) (Figure 12F). The absorption peak of MPH in UV-vis-NIR is at 200–400 nm. Observing the absorption peaks of PEG and HA, it can be found that MPH is a polymer in which PEG and HA have been mixed (Figure 12G). In order to further explore the effect of PTT, mice with lung cancer were divided into two groups: 1) 1,064 nm laser 2) 1,064 nm laser + MPH. After 10 min of laser irradiation, the tumor temperature in the second group increased by 14.2°C, indicating that MPH irradiated with 1,064 nm had a good photothermal conversion effect. Two groups of mice were observed for 16 days. By observing the resected tumors and the tumor cells stained with H and E, the smallest tumors and more dead tumor cells were found in the second group, indicating the good PTT effect of MPH (Figure 12H). Inspection of other organs of mice in the second group showed that there was no obvious damage, proving that MPH has good biocompatibility.

Glioma is an aggressive form of brain cancer derived from the neuroectoderm, and is difficulty to traet with symptoms including headache, vomiting, epilepsy, etc. The use of PTT to inhibit glioma is a promising research direction. Wen et al. designed and synthesized a thiadiazoloquinoline-based semiconducting polymer (Wen et al., 2020b), and further prepared it into SPNs through the nanoprecipitation method. The SPNs were used to treat glioma in NIR-II PAI-guided PTT (Figure 12I). There is an absorption peak of the nanoparticles at 929 nm in NIR-I, and a vibration shoulder at 1,030 nm in NIR-II. Such a wide absorption band allows phototherapy in both NIR windows (Figure 12J). NIR-II light-excited PTT against U87 glioma was performed within 24 h after SPNs injection, which is the best time point. With 1,064 nm radiation (intensity of 1.0 w cm^−2^), the local temperature of the tumor site increased significantly and reached 48 °C under 10 min of laser irradiation (Figure 12K). This temperature is sufficient to ablate tumor tissue. In contrast, the temperature of the tumor area treated with PBS +1,064 nm laser did not increase significantly, indicating that local hyperthermia is indeed the result of the combined action of SPNs and laser. The excellent tumor elimination effect of SPNs under NIR-II laser irradiation effectively improved the survival rate of mice. *In vivo* examinations showed that after intravenous injection, SPNs are easy to passively target to tumor sites including subcutaneous tumors and brain tumors, so the NIR-II laser induced PAI and PTT can target gliomas in superficial and deep tissues. This work provided an effective photothermal reagent, which broadened the structural diversity of SPN used for PTT of brain tumors. Han et al. (2018) constructed a “therapeutic mesoporous” layer on the surface of 2D Nb_2_C MXene, which not only facilitated the surface engineering of 2D MXene, but also enhances its PTT effect through adjuvant chemotherapy. The effective surface targeted modification was achieved through arginine-glycine-aspartic pentapeptide c (RGDyc) coupling, and the RGD peptides enabled higher accumulation in tumor tissues and further improved the therapeutic effect. The tumor-bearing mice and tumor tissues was monitored within 15 days after photothermal treatment and continuous chemotherapy. The tumor-bearing mice in the CTAC@Nb_2_C-MSN-polyethylene glycol-RGD group combined with laser irradiation at1064 nm showed a significantly enhanced tumor suppression and the tumors almost completely eradicated after the enhanced treatment. The tumor suppression efficiency reached 92.37%, which was much higher than the CTAC@Nb_2_C-MSN-PEG-RGD chemotherapy group (35.96%). These composite nanosheets can be gradually excreted from the body through feces and urine, and exhibited good biocompatibility. This work provided an effective strategy for the surface engineering of 2D MXenes to meet various application requirements, and also greatly broadens the biomedical applications of 2D Nb_2_C MXene in enhanced cancer treatment.

PTT based on various materials has great potential in solving major problems of lung cancer and glioma due to its non-invasive characteristics and high eficacy. Coupling with the deep penetration of NIR-II, this field is an ideal solution for clinical cancer treatment.

## Conclusion and Perspective

In recent years, the photothermal treatment using NIR-II materials, as a new cancer therapy, has developed rapidly. In this review, we summarized the photothermal properties of Au nanomaterials, two-dimensional materials, metal sulfide oxides and polymers in the NIR-II, and made a corresponding brief description of the potential applications of these materials in PTT. NIR-II materials have shown exciting application prospects, however, they are still in the initial stage and therefore faced with some challenges:

(1) At present, materials that can be applied to NIR-II PTT are still limited. More advanced synthesis methods should be explored to synthesize more materials for NIR-II PTT. Further improvements in molecular engineering may be able to break through this obstacle.
(2) Most of the materials that can be used for NIR-II PTT are inorganic nanomaterials and small molecule dyes, which have possible long-term biological toxicity. In order to improve the biocompatibility of the NIR-II material, the molecular structure of the material should be designed reasonably. Polymer materials tend to have better degradation properties, and introducing imine bonds to the molecules can improve the biodegradation. Additionally, nanomaterials can be designed to be smaller than the kidney filtration threshold (5 nm) to improve the biocompatibility of the material.
(3) Due to the physical limitation of the penetration depth of the NIR-II light, most of the light cannot reach the tumor. In order to make full use of the light in the NIR-II region, materials with higher possible absorption, extinction coefficient, and photothermal conversion efficiency should be explored to offset the energy dissipation during the irradiation of deep tissues.
(4) The specificity of materials require improvement. The NIR-II material should be precisely designed, and the specificity and targeting of the material in the organism can be improved by surface functionalization. This improvement is of great significance for more precise photothermal treatment.

In conclusion, NIR-II materials are promising in use for photothermal treatment, yet the materials need to be improved. It is foreseeable that the materials in the NIR-II will have faster development and broader application prospects in the next few years.
